# A Fluorescence Polarization Assay for Macrodomains Facilitates the Identification of Potent Inhibitors of the SARS-CoV-2 Macrodomain

**DOI:** 10.1021/acschembio.3c00092

**Published:** 2023-05-01

**Authors:** Ananya Anmangandla, Sadhan Jana, Kewen Peng, Shamar D. Wallace, Saket R. Bagde, Bryon S. Drown, Jiashu Xu, Paul J. Hergenrother, J. Christopher Fromme, Hening Lin

**Affiliations:** † Department of Chemistry and Chemical Biology, Cornell University, Ithaca, New York 14853, United States; ‡ Department of Molecular Biology and Genetics, Weill Institute for Cell and Molecular Biology, Cornell University, Ithaca, New York 14853, United States; § Department of Chemistry, Institute for Genomic Biology, and Cancer Center at Illinois, University of Illinois at Urbana-Champaign, 261 Roger Adams Lab Box 36-5, 600 S. Mathews Avenue, Urbana, Illinois 61801, United States; ∥ Howard Hughes Medical Institute; Department of Chemistry and Chemical Biology, Cornell University, Ithaca, New York 14853, United States

## Abstract

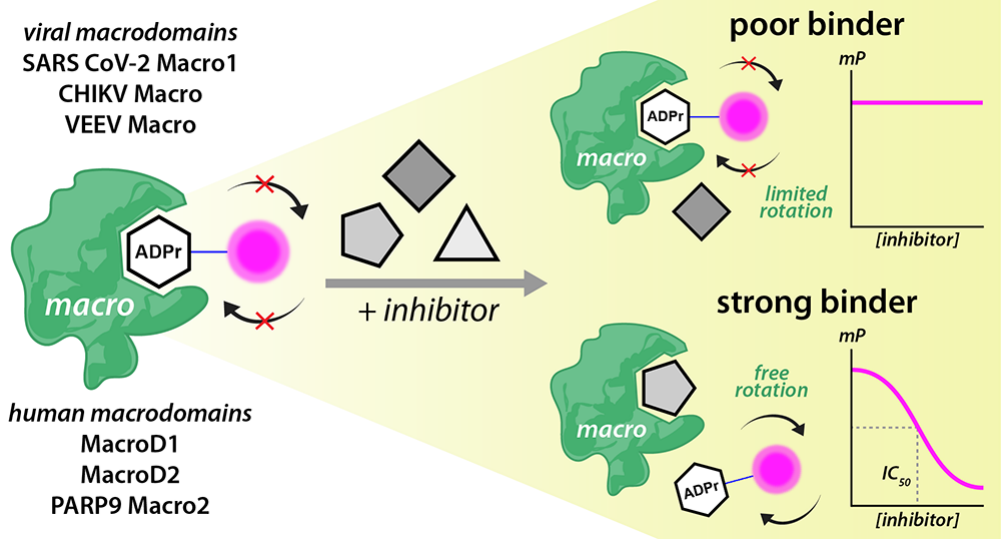

Viral macrodomains, which can bind to and/or hydrolyze adenine diphosphate ribose (ADP-ribose or ADPr) from proteins, have been suggested to counteract host immune response and be viable targets for the development of antiviral drugs. Therefore, developing high-throughput screening (HTS) techniques for macrodomain inhibitors is of great interest. Herein, using a novel tracer **TAMRA-ADPr**, an ADP-ribose compound conjugated with tetramethylrhodamine, we developed a robust fluorescence polarization assay for various viral and human macrodomains including SARS-CoV-2 Macro1, VEEV Macro, CHIKV Macro, human MacroD1, MacroD2, and PARP9 Macro2. Using this assay, we validated **Z8539** (IC_50_ 6.4 μM) and **GS441524** (IC_50_ 15.2 μM), two literature-reported small-molecule inhibitors of SARS-CoV-2 Macro1. Our data suggest that **GS441524** is highly selective for SARS-CoV-2 Macro1 over other human and viral macrodomains. Furthermore, using this assay, we identified **pNP-ADPr** (ADP-ribosylated *p*-nitrophenol, IC_50_ 370 nM) and **TFMU-ADPr** (ADP-ribosylated trifluoromethyl umbelliferone, IC_50_ 590 nM) as the most potent SARS-CoV-2 Macro1 binders reported to date. An X-ray crystal structure of SARS-CoV-2 Macro1 in complex with TFMU-ADPr revealed how the TFMU moiety contributes to the binding affinity. Our data demonstrate that this fluorescence polarization assay is a useful addition to the HTS methods for the identification of macrodomain inhibitors.

## Introduction

COVID-19 is an ongoing global pandemic caused by severe acute respiratory syndrome coronavirus 2 (SARS-CoV-2) that has led to more than 6.8 million deaths and over 759 million cases worldwide.^1^ As one of the major host defense mechanisms against viral infections, interferon (IFN) signaling is activated when host cells detect viral invasions.^2^ A central effector of IFN activation is the ADP-ribosylation of host cell proteins, and these ADP-ribose (ADPr) tags play important roles in regulating protein activities and are thus vital for a successful defense against viral infection.^3^

However, SARS-CoV-2 can counter IFN-induced mono-ADP-ribosylation (MARylation) in host cells through its first macrodomain (Macro1) encoded within the non-structural protein 3 (nsp3).^4^ Macrodomains are ancient and well-conserved structural modules found in a wide range of proteins with diverse biological functions. Macrodomains are known to bind, and in some cases, hydrolyze ADP-ribosylated proteins, thus functioning as either “readers” or “erasers” of ADPr modifications. Viral macrodomains, including those of coronaviruses (CoVs), the Venezuelan equine encephalitis virus (VEEV), and the Chikungunya virus (CHIKV), are reported to hydrolyze MARylated host proteins and are responsible for attenuating host immune responses against viral infection.^4−6^ Given the central roles that viral macrodomains play in host cell immune responses, macrodomain inhibitors are potential antiviral agents. However, several macrodomains are also encoded by human proteins, including MacroD1, MacroD2, PARP9, and TARG1, which may have important physiological functions.^7^ Therefore, selective viral macrodomain inhibitors with minimal off-target effects are highly desirable.

With the emergence of the COVID-19 global pandemic, multiple research efforts have been directed toward developing high-throughput screening (HTS) methods for the identification of SARS-CoV-2 Macro1 inhibitors. For instance, Dasovich et al.^8^ reported a luminescence-based assay termed ADPr-Glo, which utilizes an ADP-ribosylated peptide that can be hydrolyzed by SARS-CoV-2 Macro1. The hydrolysis product ADPr can be further hydrolyzed into AMP by the phosphodiesterase NudF and is subsequently converted into luminance by the commercially available AMP-Glo and quantified. Schuller et al.^9^ screened over 200 crystallographic and virtual screening hits using a homogeneous time-resolved fluorescence (HTRF) assay and differential scanning fluorimetry (DSF) assay.

These HTS assays for SARS-CoV-2 Macro1 have several limitations. For the ADPr-Glo assay, an extra enzyme NudF is used, which can complicate the result since compounds may also affect NudF activity. HTRF utilizes three expensive reagents (ADPr-conjugated biotin peptide, FRET donors and acceptors), rendering it less cost-effective for large-scale screening. Finally, the DSF assay is not suitable for high-throughput screening. More facile HTS methods have been developed for other macrodomains such as PARG,^10,11^ but they cannot be used for other macrodomains.

The fluorescence polarization (FP) assay, which exploits the polarization of a fluorophore being inversely related to its freedom of motion,^12^ provides a useful addition to the screening methods mentioned above. The FP assay is a simple and high-throughput assay that can be performed in ambient conditions, and the only reagent it requires other than the protein of interest is a fluorophore-conjugated ligand (so-called “tracer”). Very recently, Roy et al.^13^ developed an FP assay for the screening of SARS-CoV-2 Macro1 using fluorescein-labeled and ADP-ribosylated peptide as the tracer. However, they could only achieve an assay window of less than 60 milipolarization (mP) using a high protein concentration of 15 μM, which suggests that the tracer may not be a high-affinity binder of SARS-CoV-2 Macro1 and necessitates the use of large quantities of protein, limiting its use as a high-throughput screening method.

Herein, we designed and synthesized a novel FP tracer, **TAMRA-ADPr**. Using this tracer, we established a robust binding assay with a wider mP shift window and successfully applied this assay to a variety of macrodomains, including SARS-CoV-2 Macro1, VEEV Macro, CHIKV Macro, human MacroD1, MacroD2, and PARP9 Macro2. Using this assay, we were able to validate two small-molecule SARS-CoV-2 Macro1 inhibitors reported in the literature. Furthermore, we tested several ADPr derivatives and identified two compounds to be the most potent binders of SARS-CoV-2 Macro1 known to date.

## Results and Discussion

**Figure 1:**
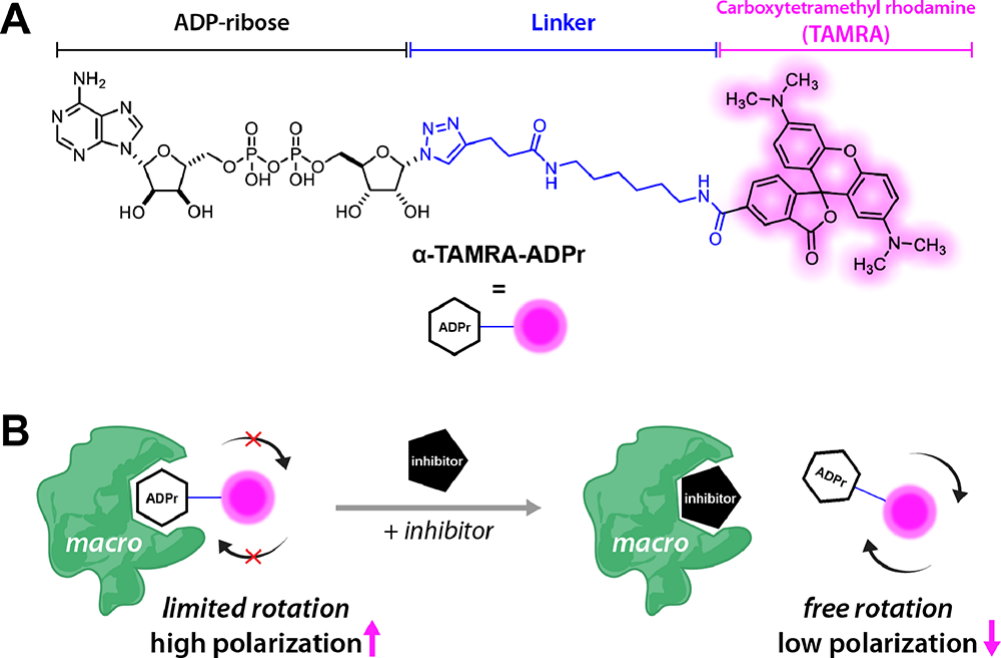
Design and mechanism of a fluorescence polarization (FP) assay for ADPr-binding macrodomains. (A) Structure of **TAMRA-ADPr**. The TAMRA fluorophore is coupled to ADPr at C1″ through a long triazole-alkane linker. (B) In the absence of inhibitors, the majority of tracers is bound to protein. Thus, the free rotation of the fluorophore is hindered and a high fluorescence polarization is observed. Upon addition of inhibitor, there is competition for binding and the tracer is released from the macrodomain. The unbound tracer molecules are now free to rotate, leading to a lower observed polarization.

**Figure 2:**
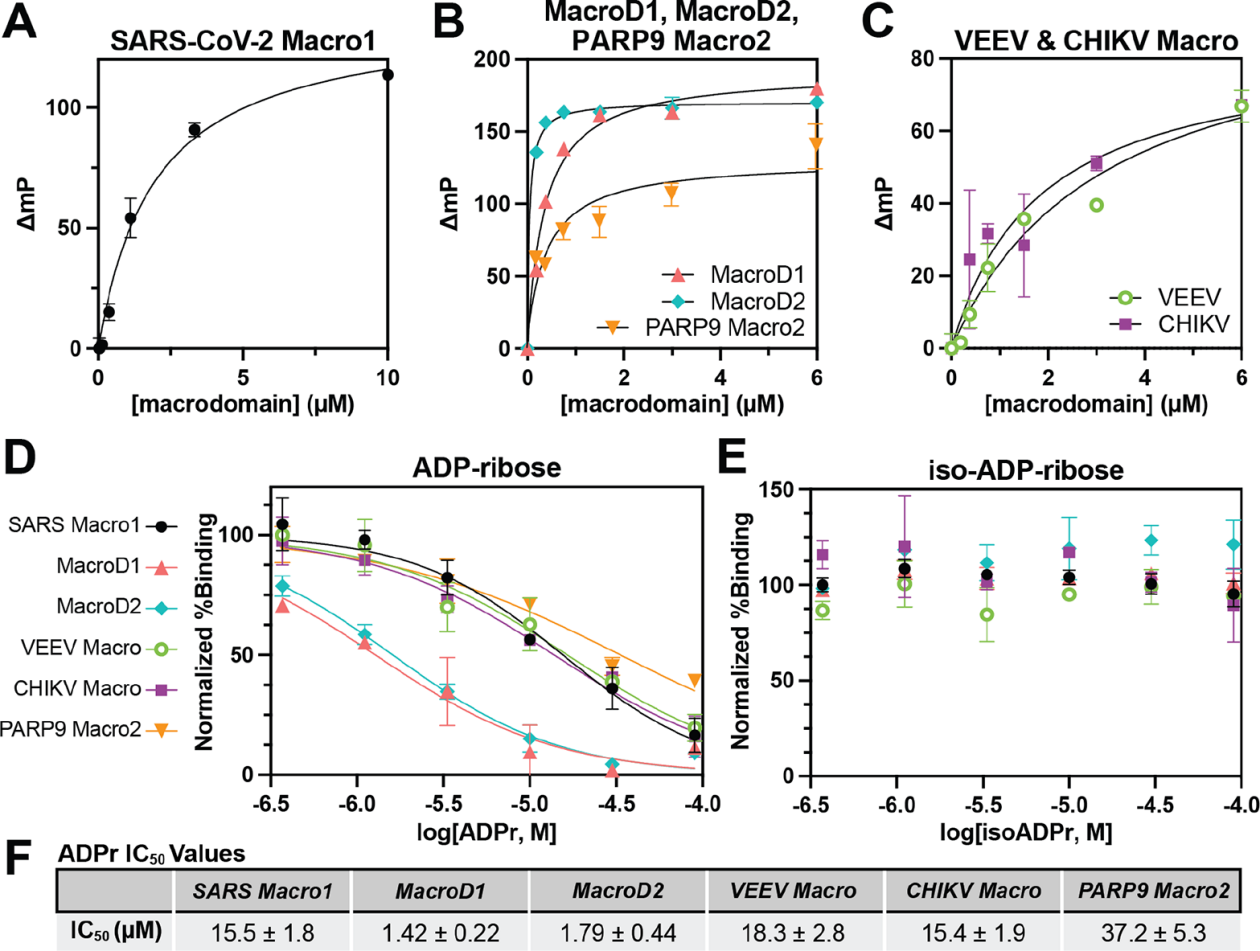
Validation of the FP assay. (A–C) mP shift values measured after 30 min incubation of 20 nM tracer with varying concentrations of macrodomains. (D) *K*_D_ curves of ADPr for different macrodomains. (E) iso-ADPr does not compete with the tracer for all the macrodomains tested. (F) IC_50_ values for ADPr with different macrodomains. For all the data presented, error bars indicate SEM and IC_50_ values are reported as mean ± SEM, *n* = 2 or 3.

To develop an FP assay suitable for the high-throughput screening of SARS-CoV-2 Macro1 inhibitors, we first designed a tracer molecule **TAMRA-ADPr** (Figure 1), inspired by the presumed structure of macrodomain substrates.^7^ For the synthesis of **TAMRA-ADPr**, **ADPr-N_3_** was first synthesized and then coupled to alkyne-TAMRA at the C1″ position via click chemistry (see the SI for details). Gratifyingly, **TAMRA-ADPr** showed relatively strong binding to SARS-CoV-2 Macro1 in a titration assay where the mP shift reached over 110 when 10 μM Macro1 was used (Figure 2A). Encouraged by this result, we tested five additional macro domains: human MacroD1, MacroD2, PARP9 Macro2, VEEV Macro, and CHIKV Macro. **TAMRA-ADPr** could bind to each of these macro domains, albeit with different affinities. Based on the mP shift data, MacroD1 and MacroD2 are the most potent binders of **TAMRA-ADPr**, reaching mP shifts of more than 100 at a low concentration of 0.38 μM (Figure 2B). This is consistent with the previous finding that ADPr is a strong binder of both MacroD1 (*K*_D_ = 0.72 μM)^14^ and MacroD2 (*K*_D_ = 0.15 μM).^15^ On the other hand, only an ∼70 mP shift could be achieved by VEEV Macro and CHIKV Macro at 6 μM (Figure 2C), suggesting that they are weaker binders of **TAMRA-ADPr**.

Having obtained a satisfactory tracer, we next designed a convenient “mix and read” FP assay where different concentrations of compounds to be tested were incubated with SARS-CoV-2 Macro1 and the tracer for 30 min before the mP shifts were read on a plate reader. The percent binding of the tracer relative to the negative control (protein and tracer only) was calculated and fitted to an IC_50_ curve. It should be noted that the protein concentrations were chosen to give an mP shift window of at least 50 to yield data with acceptable errors and therefore differ for each macrodomain (see Materials and Methods). We first tested ADPr, a well-characterized ligand for SARS-CoV-2 Macro1 as well as many other macro domains, to see whether our FP assay could quantitatively capture the binding affinity of macrodomain ligands. The IC_50_ of ADPr against the tracer binding to SARS-CoV-2 Macro1 was determined to be 15.5 μM (Figure 2D,F), which is comparable to the reported *K*_D_ value of 11.6 μM.^16^ We further determined the IC_50_ values of ADPr against other macrodomains (Figure 2D,F) and were pleased to find the IC_50_ values were all consistent with the reported *K*_D_ values of ADPr for different macrodomains.^6,14,15^ As a negative control, we also showed that iso-ADPr, the smallest internal structural unit containing the characteristic ribose–ribose glycosidic bond for poly-ADPr (PAR),^17,18^ did not compete with the tracer in macrodomain binding (Figure 2E). Therefore, we concluded that this competitive FP assay is a reliable screening method for potential inhibitors of SARS-CoV-2 Macro1 and other ADPr-binding macro domains.

**Figure 3:**
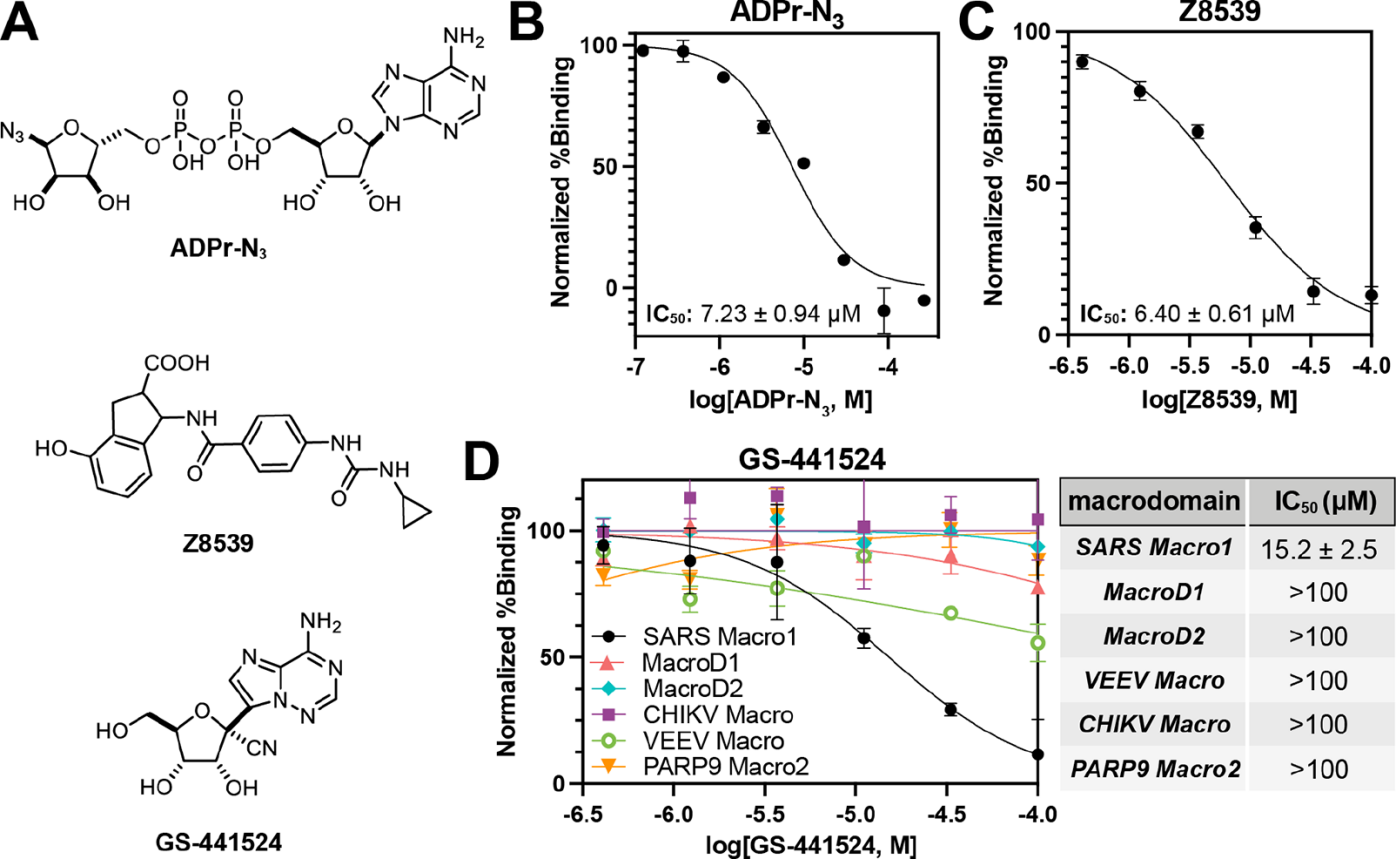
IC_50_ determination of **ADPr-N_3_, Z8539**, and **GS-441524** on SARS-CoV-2 Macro1. (A) Chemical structures of **ADPr-N_3_**, **Z8539**, and **GS-441524**. (B) IC_50_ curve of ADP-N_3_ for SARS-CoV-2 Macro1. (C) IC_50_ curve of **Z8539** for SARS-CoV-2 Macro1. (D) IC_50_ curves and values of **GS-441524** for different macrodomains. For all the data presented, error bars indicate SEM and IC_50_ values are reported as mean ± SEM, *n* = 2 or *n* = 3.

Since **TAMRA-ADPr** is a good binder of SARS-CoV-2 Macro1, we thought it would be interesting to see whether **ADPr-N_3_**, the precursor of **TAMRA-ADPr**, could also bind SARS-CoV-2 Macro1. As shown in Figure 3B, **ADPr-N_3_** was identified to be a more potent binder of SARS-CoV-2 Macro1 than ADPr with a nearly 2-fold smaller IC_50_. The binding activity of **ADPr-N_3_** is not unexpected since the azido group is similar to the hydroxyl group in size and thus unlikely to cause steric clashes with the protein. Given that SARS-CoV-2 Macro1 can accommodate much bulkier groups at the C1″ position of ADPr, as shown by **TAMRA-ADPr**, **ADPr-N_3_** may be a useful precursor for the development of ADPr-based inhibitors of SARS-CoV-2 Macro1 through click chemistry.

We then tested two recently reported SARS-CoV-2 Macro1 inhibitors using the FP assay. **Z8539** (Figure 3A) is a potent small-molecule inhibitor of SARS-CoV-2 Macro1 discovered very recently by Gahbauer et al.^19^ through a combined approach of virtual screening and fragment linking. **Z8539** was found to be a slightly better SARS-CoV-2 Macro1 binder than ADPr in a homogeneous time-resolved fluorescence (HTRF) assay. This result was validated in our FP assay, where the IC_50_ of **Z8539** is 2-fold smaller than ADPr against SARS-CoV-2 Macro1 (Figure 3C). **Z8539** is an encouraging example, showing that small-molecule inhibitors of SARS-CoV-2 Macro1 with structures unrelated to ADPr are possible. However, its binding affinity for SARS-CoV-2 Macro1 is only ∼6.4 μM.

Another reported small-molecule inhibitor of SARS-CoV-2 Macro1 is **GS-441524** (Figure 3A), the active metabolite of Remdesivir, an antiviral drug that targets the viral RNA-dependent RNA polymerase (RdRp).^20^ Remdesivir was shown to be effective against SARS-CoV-2^21^ and is the first COVID-19 therapy approved by the FDA. Recently, Ni et al.^22^ found that **GS-441524** can bind SARS-CoV-2 Macro1 and solved the crystal structure of SARS-CoV-2 Macro1 bound with **GS-441524**. Using isothermal titration calorimetry (ITC), they determined that the *K*_D_ of **GS-441524** for SARS-CoV-2 Macro1 is 10.8 μM, similar to that of ADPr. Intrigued by this finding, we also tested **GS-441524** in our FP assay. Consistent with the reported data, the IC_50_ of **GS-441524** for SARS-CoV-2 Macro1 was determined to be 15.2 μM. Additionally, we tested whether **GS-441524** could inhibit other macrodomains and were surprised to find that **GS-441524** is a selective SARS-CoV-2 Macro1 inhibitor with no significant binding to all other macrodomains tested (Figure 3D). This result coincides with another paper published very recently,^23^ which showed that **GS-441524** is selective for SARS-CoV-2 Macro1 over other macrodomains including MERS-CoV Mac, CHIKV Macro, PARP14 Macro2, and PARP15 Macro2 in ITC experiments. Taken together, **GS-441524** is a promising lead compound against SARS-CoV-2 Macro1 with high selectivity and ligand efficiency.

**Figure 4:**
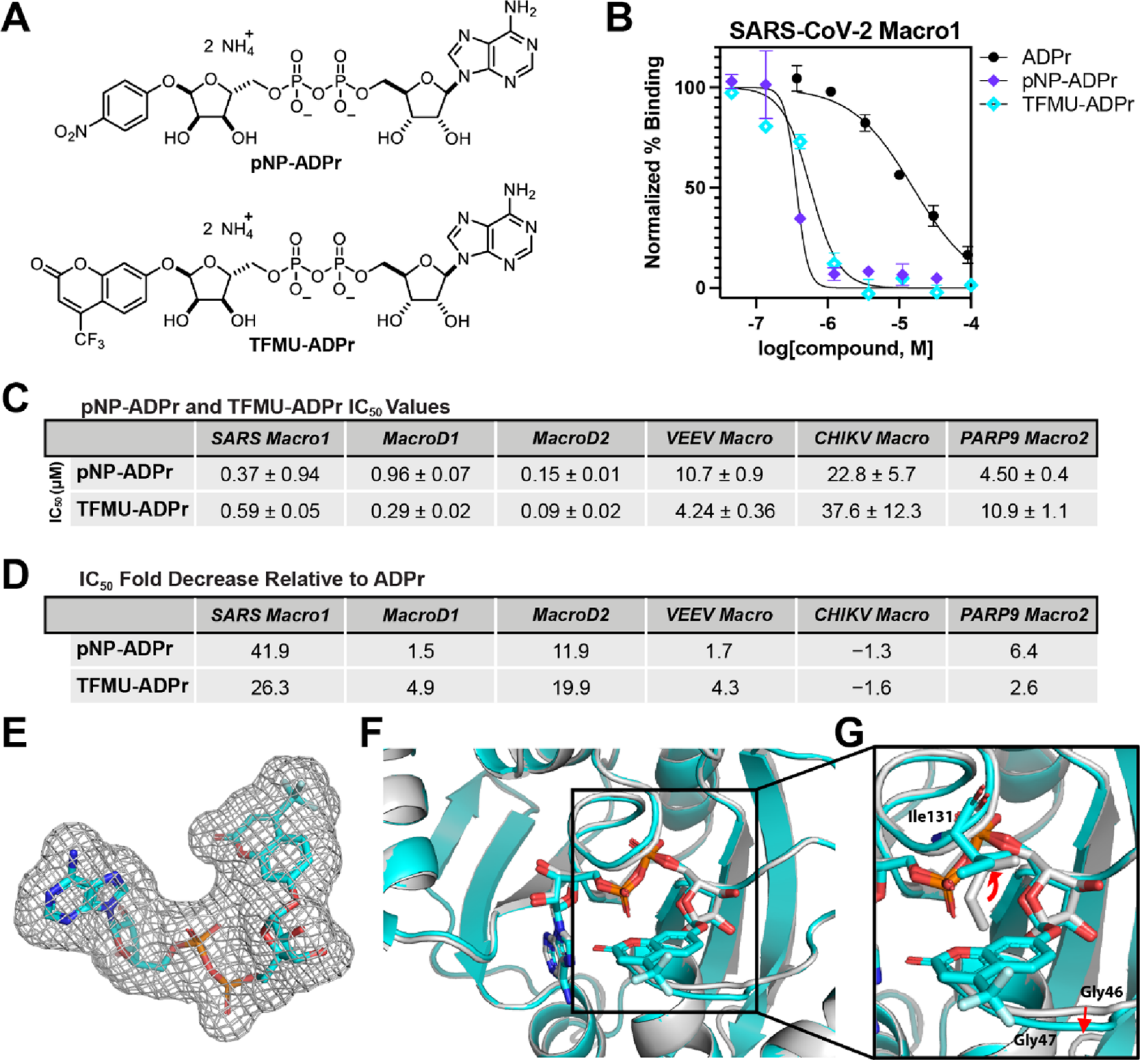
**pNP-ADPr** and **TFMU-ADPr** are potent macrodomain binders. (A) Chemical structures of **pNP-ADPr** and **TFMU-ADPr**. (B) IC_50_ curves of **pNP-ADPr** and **TFMU-ADPr** for SARS-CoV-2 Macro1. IC_50_ curve of ADPr is also shown as a reference. (C) IC_50_ values of **pNP-ADPr** and **TFMU-ADPr** for SARS-CoV-2 Macro1. (D) Fold decrease in the IC_50_ values of **pNP-ADPr** and **TFMU-ADPr** relative to those of ADPr for each macrodomain tested. (E) Electron density of **TFMU-ADPr** in the complex with SARS-CoV-2 Macro1. (F) Structure of Macro1 in complex with **TFMU-ADPr** (cyan) is superimposed with that of Macro1 in complex with ADPr (gray, PDB 6YWL). **TFMU-ADPr** and ADPr are shown in stick representation. (G) Aromatic ring of TFMU interacts with the side chain of Ile131 and on the other side, Gly46 and Gly47. For all the data presented, error bars indicate SEM and IC_50_ values are reported as mean ± SEM, *n* = 2 or 3.

Although the actual physiological substrates/binding partners for SARS-CoV-2 Macro1 are still unknown, it has been proposed that the most likely substrates are ADPr C1″-esters coupled to glutamic or aspartic acid protein residues.^24^ Taken together with our finding that **TAMRA-ADPr** with a C1″-triazole linkage can bind SARS-CoV-2 Macro1 with high affinity, it seems that bulky groups at the C1″ position would not disrupt binding and instead may confer a higher affinity. We therefore tested several other ADPr compounds. **TFMU-ADPr** and **pNP-ADPr** (Figure 4A) are previously developed assay substrates for poly(ADP-ribosyl)glycohydrolase (PARG).^25^ Based on our proposal that bulkier substituents at the C1″ position may boost macrodomain binding, **TFMU-ADPr** and **pNP-ADPr** may be potential binders for macrodomains since the aromatic rings are introduced at C1″ similar to **TAMRA-ADPr**. Therefore, we tested these two compounds in the FP assay. We were surprised to find that **pNP-ADPr** is 40-fold more potent than ADPr for SARS-CoV-2 Macro1 with an IC_50_ of only 0.37 μM (Figure 4B–E), which is the strongest binder of SARS-CoV-2 Macro1 reported so far. Similarly, the IC_50_ of **TFMU-ADPr** against SARS-CoV-2 Macro1 is 0.59 μM, 25-fold smaller than ADPr (Figure 4B–E). We also tested these two compounds with other macrodomains and found that their IC_50_ values are similar to those of ADPr with the exception of MacroD2, for which both compounds showed a more than 10-fold increase in activity over ADPr (Figure 4C,D). Therefore, **pNP-ADPr** and **TFMU-ADPr** are both potent binders of macrodomains with strong preferences for SARS-CoV-2 Macro1. We also found that **pNP-ADPr** and **TFMU-ADPr** could inhibit the hydrolysis of ADPr by SARS-CoV-2 Marco1 and human MacroD1 in the cell lysate (Figure S1, Supporting Information).

To understand why TFMU-ADPr binds strongly to Macro1, we determined the X-ray crystal structure of the Macro1-TFMU-ADPr complex using diffraction data that extended to 1.9 Å resolution. The TFMU-ADPr electron density is well resolved (Figure 4E). As expected, the inhibitor binds within the known ADPr binding site of Macro1 (Figure 4F, the structure is superimposed to that of the Macro1-ADPr complex PDB 6YWL). The TFMU moiety extends from the binding site along a narrow hydrophobic groove, bracketed on one side by the Ile131 side chain and on the other side by Gly46 and Gly47 (Figure 4G).

## Conclusions

In summary, we have developed **TAMRA-ADPr**, an ADPr-based tracer, and devised an FP-based binding assay for the screening of ADPr-binding macrodomain inhibitors. The reliability of the FP assay was confirmed by testing the IC_50_ values of ADPr against different macrodomains and comparing them to the reported *K*_D_ values. Using this assay, we tested and validated **Z8539** and **GS-441524**, two recently reported small-molecule inhibitors of SARS-CoV-2 Macro1. An interesting finding of this work is that **pNP-ADPr** and **TFMU-ADPr** are strong binders of SARS-CoV-2 Macro1 and several other macrodomains. Their structures may provide clues for the future design of more potent ADPr-based probe molecules of SARS-CoV-2 Macro1. We believe that the FP assay described herein is a convenient and robust screening method that can facilitate future drug discovery efforts for macrodomain inhibitors.

## Materials and Methods

### Reagents

**pNP-ADPr** and **TFMU-ADPr** are synthesized as previously described.^25^**Z8539** was obtained from Enamine (Z4718398539). **GS-441524** was obtained from MedChemExpress (HY-103586).

### Expression and Purification of Macrodomains

Macrodomain plasmids were purchased from Twist Biosciences or Genscript in pET28 vectors (full sequences available in the SI). The plasmids were transformed into BL21(DE3) chemically competent *E. coli*. 4 L of LB broth with 50 μg/mL kanamycin was inoculated with an overnight starter grown at 37 °C. Cultures were grown at 200 rpm and 37 °C for ∼4 h until the OD600 reached 0.8. Then, IPTG was added to 0.5 mM and the cells were incubated at 16 ° C overnight to allow protein expression. Cells were harvested by centrifugation at 6000*g*. Cell pellets were frozen at −80 °C or immediately used for purification. Pellets were resuspended in lysis buffer (50 mM Tris pH 8.0, 500 mM NaCl, 0.5 mg/mL lysozyme, 1 mM PMSF, and Pierce universal nuclease). Following a 30 min incubation, cells were sonicated on ice for 4 min total at 60% amplitude. The lysate was clarified at 4 °C and 30,000*g* for 35 min. The clarified lysate was loaded onto Ni-NTA resin, washed with 50 mL wash buffer (50 mM Tris pH 8.0, 500 mM NaCl, 20 mM imidazole), and eluted with elution buffer (50 mM Tris pH 8, 500 mM NaCl, 200 mM imidazole). Crude macrodomains were concentrated using a 10 kDa MWCO Amicon filter and loaded onto a HiLoad 16/600 Superdex 75 gel filtration column equilibrated with storage buffer (25 mM Tris pH 8.0, 150 mM NaCl, 10% glycerol) on an ÄKTA FPLC system. Fractions containing macrodomains were pooled, concentrated, flash-frozen in liquid nitrogen, and stored at −80 °C for future use. For SARS-CoV-2, the sample was supplemented with DTT (2 mM) and tobacco-etch protease and incubated at 4 °C overnight. The reaction mixture was then subjected to subtractive nickel chelate chromatography, and the eluate was injected into a HiLoad 16/600 Superdex75 gel filtration column equilibrated with protein storage buffer (5 mM HEPES and 150 mM NaCl, pH 7.5). Fractions containing purified the SARS-CoV-2 macrodomain were combined and concentrated. Then, samples were aliquoted, flash frozen using liquid nitrogen, and stored at −80 °C.

### Fluorescence Polarization Assay

The stock solution of purified macrodomain proteins was diluted with the assay buffer (25 mM Tris pH 8.0, 150 mM NaCl, and 0.01% Tween-20) to 2× final concentration. Final concentrations for each macrodomain were as follows: 0.5 μM for MacroD1 and MacroD2, 1.5 μM for SARS-CoV-2 Macro1 and PARP9 Macro2, and 5 μM for VEEV Macro and CHIKV Macro. TAMRA-ADPr (40 nM, 2× final concentration) was then added to the protein solution to give the assay solution. To each well of a 96-well black plate (Corning, #3915) was added 50 μL of the assay solution followed by the addition of 50 μL of the compound solution (2× final concentration) in the assay buffer. The plate was wrapped with aluminum foil and left at room temperature for 30 min. The plate was then scanned on a Cytation5 using a FP filter cube (Agilent, part number: 8040562, Ex: 530/25, Em: 590/35). The parallel and perpendicular fluorescence intensities of each well were recorded, and the mP values were then calculated based on the blank-subtracted data. Control wells include tracer-only wells where only 20 nM tracers were present and negative-control wells where only an appropriate concentration of macrodomain protein and 20 nM tracers were present. The percent binding of tracer relative to the control wells was calculated as follows:
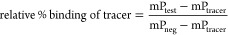
where mP_test_, mP_tracer_, and mP_neg_ are mP values of the test wells, tracer-only wells, and negative-control wells, respectively. The obtained data were then fitted into an IC_50_ curve using the sigmoidal four-parameter logistic model (bottom and top were constrained to be 0 and 100, respectively) implemented in GraphPad Prism 9.4.1 (GraphPad Software, Inc.).

### Co-crystallization of the SARS-CoV-2 Macro1-TFMU-ADPr Complex

SARS-CoV-2 Macro1 was mixed with **TFMU-ADPr** to final concentrations of 0.4 and 2 mM. The Macro1-inhibitor complex was crystallized by the hanging-drop method at 20 °C by mixing 1 μL of the Macro1-TFMU-ADPr solution with 1 μL well solution (200 mM sodium acetate, 100 mM Tris–HCl pH 8, and 30% PEG-4000). Crystals were observed after 5 days. Prior to freezing with liquid nitrogen, crystals were cryo-protected in well solution containing 10% ethylene glycol.

### Diffraction Data Collection, Structure Solution, Model Building, and Refinement

Diffraction data was collected on Northeastern Collaborative Access Team (NE-CAT) beamline 24-ID-E at Advanced Photon Source (APS). Initial data processing was performed by the NE-CAT ‘RAPD’ pipeline, which uses XDS for scaling and merging.^26^ The structure was solved by molecular replacement using Phaser^27^ in Phenix^28^ using a previously published structure of SARS-CoV-2 Macro1^22^ (PDB:6YWL) as the search model. Coot^29^ was used for model building, and refinement and validation were performed in Phenix.^30^ There are two copies of the Macro1-inhibitor complex in the asymmetric unit, so non-crystallographic symmetry restraints were used during refinement. The final structures of the two copies are nearly identical, with no obvious differences in the inhibitor or inhibitor binding sites between the two copies, although the density for the inhibitor was stronger in one copy than the other. Data and refinement statistics are presented in Table 1.

**Table 1:** Data Collection and Refinement Statistics^a^

PDB code	8GIA
resolution range	68.92–1.86 (1.926–1.86)
space group	C 1 2 1
unit cell	140.517 Å 36.668 Å 65.056 Å
	90° 101.211° 90°
total reflections	186,998 (18900)
unique reflections	27,584 (2723)
multiplicity	6.8 (6.9)
completeness (%)	99.15 (99.02)
mean *I*/sigma(*I*)	6.94 (1.36)
Wilson B-factor	28.01
*R*-merge	0.1788 (1.245)
*R*-meas	0.1943 (1.347)
*R*-pim	0.07503 (0.5084)
CC1/2	0.987 (0.704)
CC*	0.997 (0.909)
reflections used in refinement	27,525 (2717)
reflections used for *R*-free	1353 (146)
*R*-work	0.2083 (0.3189)
*R*-free	0.2586 (0.3661)
CC(work)	0.938 (0.828)
CC(free)	0.918 (0.743)
number of non-hydrogen atoms	2813
macromolecules	2535
ligands	150
solvent	176
protein residues	335
RMS (bonds)	0.005
RMS (angles)	0.60
Ramachandran favored (%)	97.89
Ramachandran allowed (%)	2.11
Ramachandran outliers (%)	0.00
Rotamer outliers (%)	0.36
Clashscore	7.90
average B-factor	35.84
macromolecules	35.81
ligands	30.80
solvent	39.19
number of TLS groups	12

a Statistics for the highest-resolution shell are shown in parentheses.

## Supporting Information Available

TFMU-ADPr and pNP-ADPr inhibition of macrodomain enzymatic activity, supplementary methods, and NMR spectra of compounds (PDF)
