# Correction to “A Fluorescence Polarization Assay for Macrodomains Facilitates the Identification of Potent Inhibitors of the SARS-CoV-2 Macrodomain”

**DOI:** 10.1021/acschembio.4c00159

**Published:** 2024-03-27

**Authors:** Ananya Anmangandla, Sadhan Jana, Kewen Peng, Shamar D. Wallace, Saket R. Bagde, Bryon S. Drown, Jiashu Xu, Paul J. Hergenrother, J. Christopher Fromme, Hening Lin

We recently noticed two mistakes in the published article. In Figure 1, the structure of TAMRA was drawn incorrectly (the position of one the dimethylamino groups was wrong). In Figure 3, the structure of GS441524 contained an extra nitrogen atom. The corrected Figure 1 and Figure 3 are given below. We are sorry for this oversight and any inconvenience it may have caused. The conclusions of the article were not affected by these structure drawing errors.

**Figure 1:**
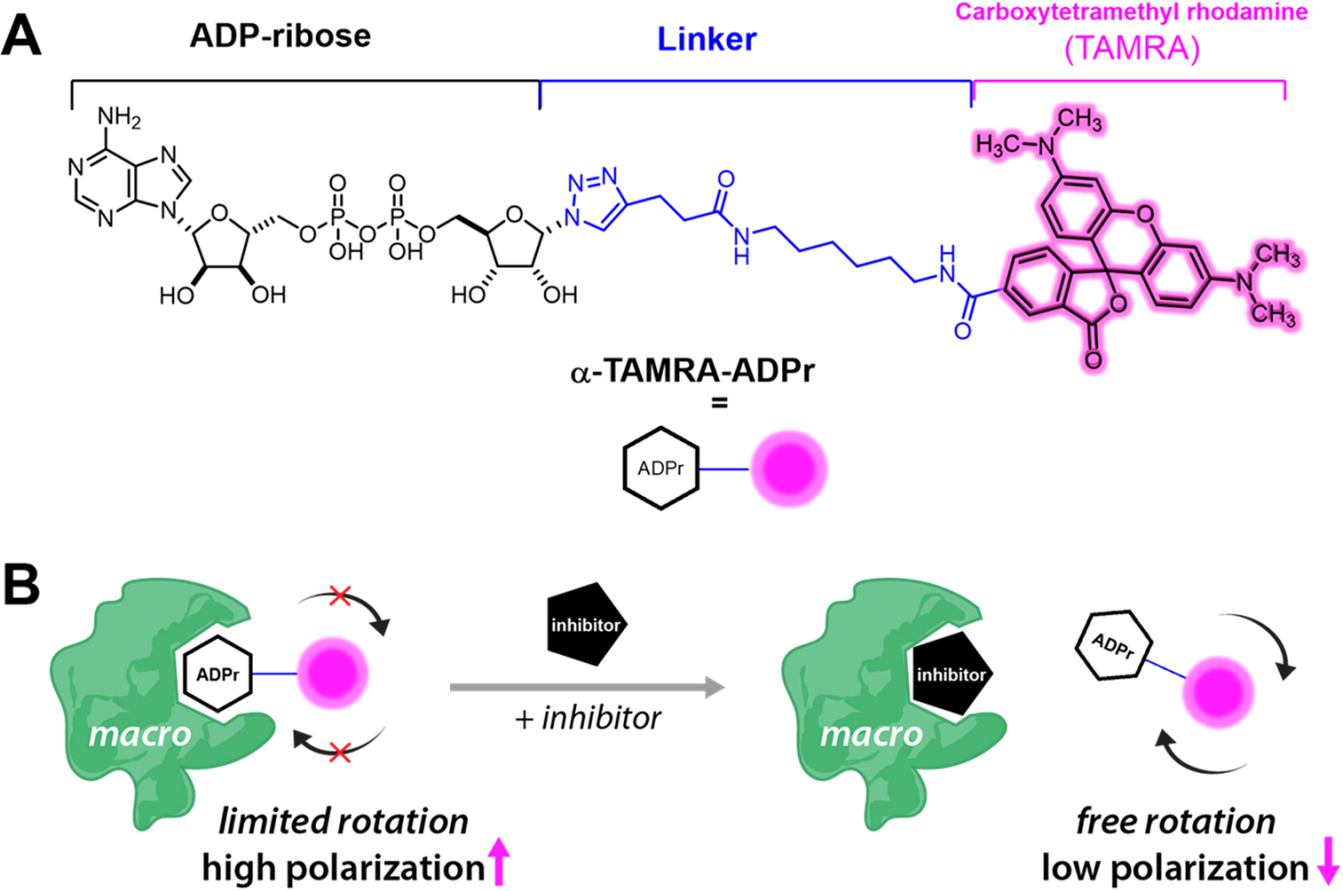
Design and mechanism of a fluorescence polarization (FP) assay for ADPr-binding macrodomains. (A) Structure of **TAMRA-ADPr**. The TAMRA fluorophore is coupled to ADPr at C1″ through a long triazole-alkane linker. (B) In the absence of inhibitors, the majority of tracers are bound to protein. Thus, the free rotation of the fluorophore is hindered and a high fluorescence polarization is observed. Upon addition of inhibitor, there is competition for binding and the tracer is released from the macrodomain. The unbound tracer molecules are now free to rotate, leading to a lower observed polarization.

**Figure 3:**
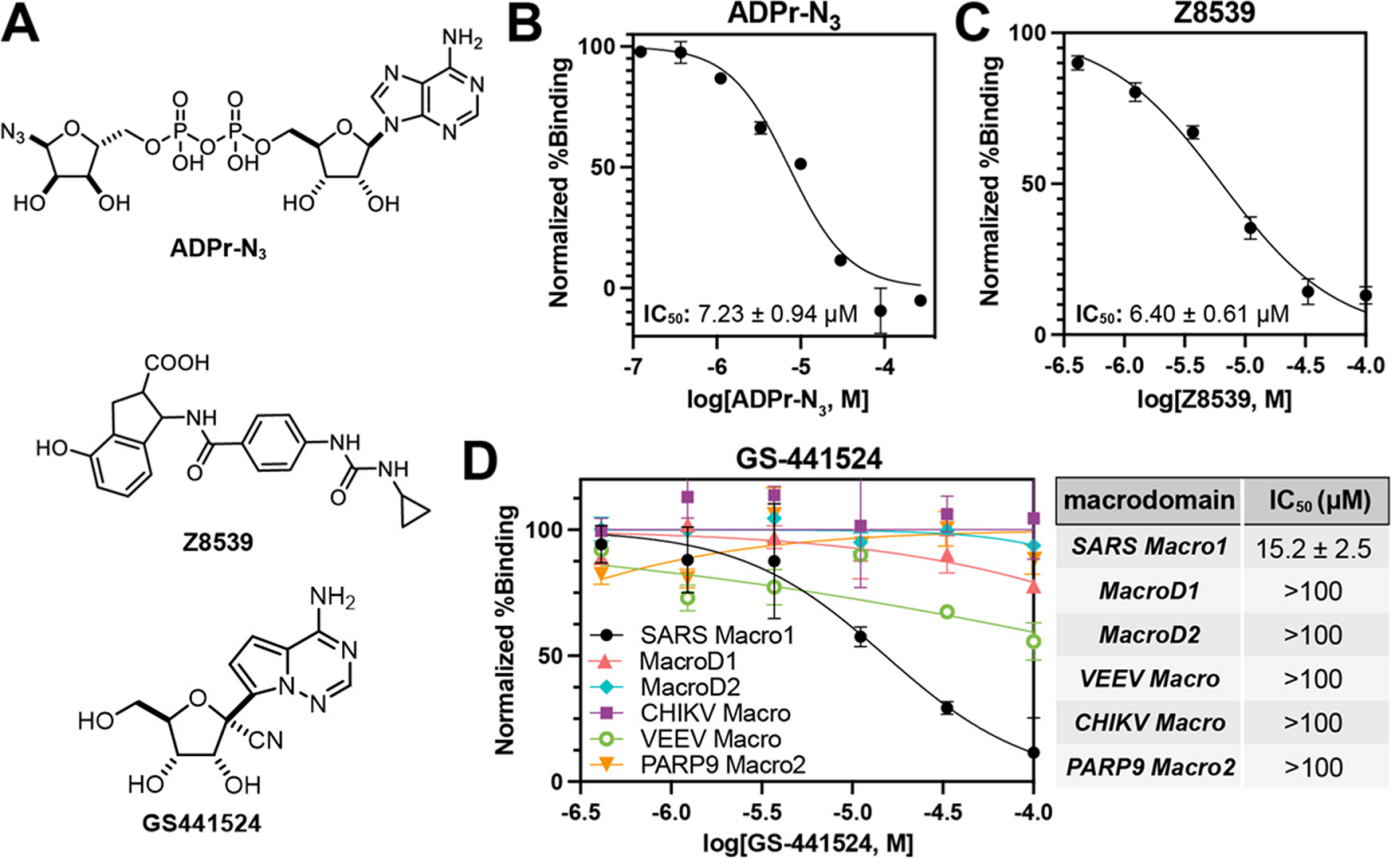
IC_50_ determination of **ADPr-N**_**3**_, **Z8539**, and **GS-441524** on SARS-CoV-2 Macro1. (A) Chemical structures of **ADPr-N**_**3**_, **Z8539**, and **GS-441524**. (B) IC_50_ curve of ADP-N_3_ for SARS-CoV-2 Macro1. (C) IC_50_ curve of **Z8539** for SARS-CoV-2 Macro1. (D) IC_50_ curves and values of **GS-441524** for different macrodomains. For all the data presented, error bars indicate SEM and IC_50_ values are reported as mean ± SEM, *n* = 2 or *n* = 3.

