# Supplementary material for: A Fluorescence Polarization Assay for Macrodomains Facilitates the Identification of Potent Inhibitors of the SARS-CoV-2 Macrodomain
Source: ACS Chem Biol. 2023 May 1;18(5):1200–7. doi: 10.1021/acschembio.3c00092 (PMC10178785; doi:10.1021/acschembio.3c00092)
Supplement: Supplementary file 1 — cb3c00092_si_001.pdf [file cb3c00092_si_001.pdf]

Supporting Information

A Fluorescence polarization assay for macrodomains facilitates the identification of potent inhibitors of SARS-CoV-2 macrodomain

Ananya Anmangandla[1, #], Sadhan Jana[1, #], Kewen Peng[1, #], Shamar D. Wallace[2,#], Saket R. Bagde[2], Bryon S. Drown[3,4], Jiashu Xu[1], Paul J. Hergenrother[3], J. Christopher Fromme[2,*], Hening Lin[1,5,*]

[1]Department of Chemistry and Chemical Biology, Cornell University, Ithaca, NY 14853, USA

[2]Department of Molecular Biology and Genetics, Weill Institute for Cell and Molecular Biology, Cornell University, Ithaca, NY 14853, USA

[3]Department of Chemistry, Institute for Genomic Biology, and Cancer Center at Illinois, University of Illinois at Urbana-Champaign, 261 Roger Adams Lab Box 36-5, 600 S. Mathews Avenue, Urbana, IL 61801, USA

[4]Current address: Department of Chemistry, Purdue University, West Lafayette, IN 47906, USA

[5]Howard Hughes Medical Institute; Department of Chemistry and Chemical Biology, Cornell University, Ithaca, NY 14853, USA

#Equal contribution

*Correspondence: hl379@cornell.edu and jcf14@cornell.edu

Supplementary Figure

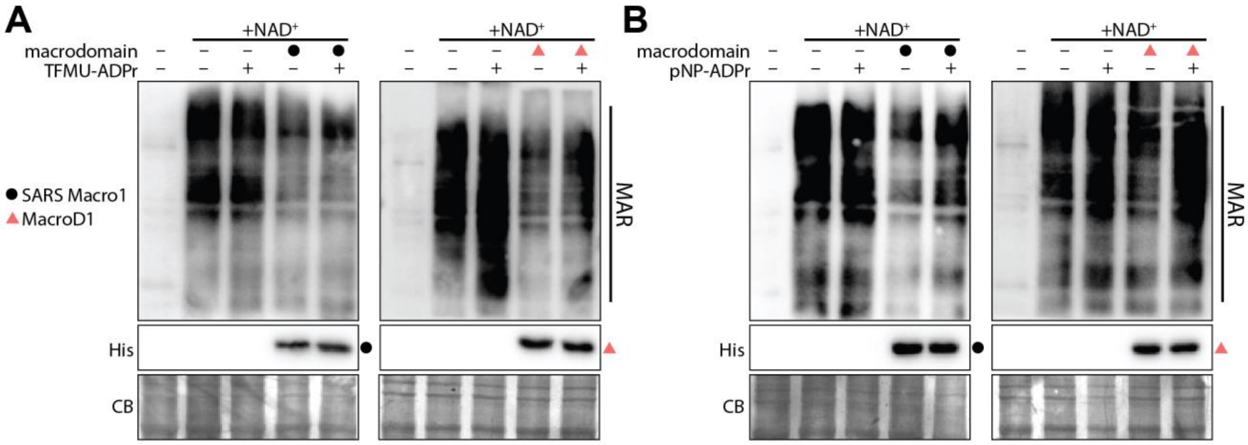

Figure S1: TFMU-ADPr and pNP-ADPr inhibit macrodomains enzymatic activity. HEK 293T cell lysates were incubated with 1 mM NAD^+ overnight to produce ADP-ribosylated protein samples. The samples were incubated with 5 µM SARS Macro1(●) or MacroD1(▲) for 2.5 hr in the presence or absence of 25 µM inhibitor. Samples were analyzed via SDS-PAGE and blotted for mono-ADPr (MAR, Sigma Aldrich MABE1076) and HRP-conjugated secondary antibody. Equal lysate loading was determined by Coomassie Blue (CB) staining. The corresponding blots show (**A**) SARS Macro1 and MacroD1 have lowered MAR hydrolysis activity in the presence of TFMU-ADPr. (**B**) SARS Macro1 and MacroD1 have lowered MAR hydrolysis activity in the presence of pNP-ADPr.

Supplementary Methods

Design of macrodomain plasmids

CHIKV Macro, VEEV Macro, PARP9 Macro2, MacroD1, and MacroD2 plasmids were designed as indicated below and purchased from Twist Biosciences. SARS-CoV-2 Macro1 was designed as indicated below and purchased from Genscript. CHIKV Macro, VEEV Macro, PARP9 Macro2, MacroD1, and MacroD2 were cloned into pET28a using NdeI and XhoI cut sites with a TEV cleavage site (gagaacctgtacttccaatcc) added to the 5' end of all inserts. For SARS-CoV-2 Macro1 a codon-optimized gene encoding the macrodomain was cloned into pET28a using NcoI and NotI restriction sites. All final constructs used in the assay contain the uncleaved 6xHis tag.

CHIKV Macro (aa1-160 of macro) (JF274082.1/M9VZ69)

MGSSHHHHHHSSGLVPRGSHMENLYFQSAPSYRVKRMDIAKNDEECVVNAANPRGLPGDGVCKAVYKKWPESFKNSA
TPVGTAKTVMCGTYPVIHAVGPNFSNYSESEGDRELAAAYREVAKEVTRLGVNSVAIPLLSTGVYSGGKDRLTQSLN
HLFTAMDSTDADVVIYCRDKEWEKKISEAIQMRT*

atgggcagcagccatcatcatcatcatcacagcagcggcctggtgccgcgcggcagccatatggagaacctgtactt
ccaatccGCTCCATCTTATCGCGTAAAGCGTATGGACATCGCTAAAAATGACGAAGAATGTGTGGTTAACGCGGCGA
ATCCGCGCGGCCTGCCAGGCGACGGGGTCTGTAAAGCGGTATATAAGAAGTGGCCTGAGAGTTTCAAAAACTCGGCC
ACTCCTGTAGGGACTGCCAAGACTGTAATGTGTGGAACATACCCTGTCATCCATGCGGTAGGACCTAATTTCTCGAA
TTACAGTGAGAGCGAGGGCGACCGTGAATTGGCGGCGGCGTACCGCGAGGTGGCCAAAGAAGTGACTCGCCTTGGCG
TGAATTCAGTAGCGATCCCCTTGCTTTCTACGGGGGTCTATAGTGGGGGCAAGGACCGTTTAACCCAAAGTCTTAAC
CATCTTTTTACTGCTATGGACTCCACAGATGCCGACGTAGTCATTTACTGCCGTGACAAGGAATGGGAAAAAAAGAT
CAGCGAAGCTATTCAGATGCGCACATGATGA

VEEV Macro (aa1-160 of macro) (L04653.1/Q8V294)

MGSSHHHHHHSSGLVPRGSHMENLYFQAPSYHVVRGDIATATEGVIINAANSKGQPGGGVCGALYKKFPESFDLQPI
EVGKARLVKGAAKHIIHAVGPNFNKVSEVEGDKQLAEAYESIAKIVNDNNYKSVAIPLLSTGIFSGNKDRLTQSLNH
LLTALDTTDADVAIYCRDKKWEMTLKEAVARRE*

atgggcagcagccatcatcatcatcatcacagcagcggcctggtgccgcgcggcagccatatggagaacctgtactt
ccaatccGCACCCTCATATCATGTGGTGCGAGGGGATATTGCCACGGCCACCGAAGGAGTGATCATAAATGCTGCTA
ACAGCAAAGGACAACCTGGCGGAGGGGTGTGCGGAGCGCTGTATAAGAAATTCCCGGAAAGCTTCGATTTACAGCCG
ATCGAAGTAGGAAAAGCGCGACTGGTCAAAGGTGCAGCTAAACATATCATTCATGCCGTAGGACCAAACTTCAACAA
AGTTTCGGAAGTTGAAGGGGACAAACAGTTGGCAGAGGCTTATGAGTCCATCGCTAAAATTGTCAACGATAACAATT
ACAAGTCAGTAGCGATTCCACTGTTGTCCACCGGCATCTTTTCCGGGAACAAAGATCGACTAACCCAATCATTGAAC
CATTTGCTGACAGCTTTAGACACCACTGATGCAGATGTAGCCATATACTGCAGGGACAAGAAATGGGAAATGACTCT
CAAGGAAGCAGTGGCTAGGAGAGAATGATGA

PARP9 Macro2 (aa306-493) (NM_031458.3/Q8IXQ6)

MGSSHHHHHHSSGLVPRGSHMENLYFQTPSFNAMVVNNLTLQIVQGHIEWQTADVIVNSVNPHDITVGPVAKSILQQ
AGVEMKSEFLATKAKQFQRSQLVLVTKGFNLFCKYIYHVLWHSEFPKPQILKHAMKECLEKCIEQNITSISFPALGT
GNMEIKKETAAEILFDEVLTFAKDHVKHQLTVKFVIFPTDLEIYKAFSSEMAKRSKMLSLN*

atgggcagcagccatcatcatcatcatcacagcagcggcctggtgccgcgcggcagccatatggagaacctgtactt
ccaatccACCCCTTCTTTCAATGCAATGGTCGTGAACAACCTGACCCTCCAGATTGTCCAGGGCCACATTGAATGGC
AGACGGCAGATGTAATTGTTAATTCTGTAAACCCACATGATATTACAGTTGGACCTGTGGCAAAGTCAATTCTACAA
CAAGCAGGAGTTGAAATGAAATCGGAATTTCTTGCCACAAAGGCTAAACAGTTTCAACGGTCCCAGTTGGTACTGGT

CACAAAAGGATTTAACTTGTTCTGTAAATATATATACCATGTACTGTGGCATTCAGAATTTCCTAAACCTCAGATAT
TAAAACATGCAATGAAGGAGTGTTTGGAAAAATGCATTGAGCAAAATATAACTTCCATTTCCTTTCCTGCCCTTGGG
ACTGGAAACATGGAAATAAAGAAGGAAACAGCAGCAGAGATTTTGTTTGATGAAGTTTTAACATTTGCCAAAGACCA
TGTAAAACACCAGTTAACTGTAAAATTTGTGATCTTTCCAACAGATTTGGAGATATATAAGGCTTTCAGTTCTGAAA
TGGCAAAGAGGTCCAAGATGCTGAGTTTGAACTGATGA

MacroD1 (aa91-325) (BC000270.1/Q9BQ69)

MGSSHHHHHHSSGLVPRGSHMENLYFQTSTDWKEAKSFLKGLSDKQREEHYFCKDFVRLKKIPTWKEMAKGVAVKVE
EPRYKKDKQLNEKISLLRSDITKLEVDAIVNAANSSLLGGGGVDGCIHRAAGPLLTDECRTLQSCKTGKAKITGGYR
LPAKYVIHTVGPIAYGEPSASQAAELRSCYLSSLDLLLEHRLRSVAFPCISTGVFGYPCEAAAEIVLATLREWLEQH
KDKVDRLIICVFLEKDEDIYRSRLPHYFPVA*

atgggcagcagccatcatcatcatcatcacagcagcggcctggtgccgcgcggcagccatatggagaacctgtactt
ccaatccACCTCCACCGACTGGAAGGAGGCGAAATCCTTTCTGAAGGGCCTGAGTGACAAGCAGCGGGAGGAACATT
ACTTCTGCAAGGACTTTGTCAGGCTGAAGAAGATCCCGACATGGAAGGAGATGGCGAAAGGGGTGGCTGTGAAGGTG
GAGGAGCCCAGGTATAAAAAGGACAAGCAGCTCAATGAGAAAATCTCCCTGCTCCGCAGCGACATCACCAAGCTGGA
GGTGGACGCCATCGTCAACGCCGCCAACAGCTCCCTGCTCGGAGGCGGTGGCGTGGACGGCTGCATTCATCGGGCCG
CCGGCCCCCTGCTTACCGACGAGTGCCGGACCCTGCAGAGCTGTAAGACTGGCAAGGCCAAGATCACCGGCGGCTAT
CGGCTCCCGGCCAAGTACGTCATCCACACAGTGGGGCCCATCGCCTACGGGGAGCCCAGCGCCAGCCAGGCTGCCGA
GCTCCGCAGCTGCTACCTGAGCAGTCTGGACCTGCTGCTGGAGCACCGGCTCCGCTCGGTGGCGTTCCCCTGCATCT
CCACCGGCGTGTTTGGCTACCCCTGTGAGGCGGCCGCCGAGATCGTGCTGGCCACGCTGCGAGAGTGGCTGGAGCAG
CACAAGGACAAGGTGGACCGGCTGATCATCTGCGTGTTCCTCGAGAAGGACGAGGACATCTACCGGAGCCGGCTCCC
CCACTACTTCCCCGTGGCCTGATGA

MacroD2 (aa10-242) (NM_001351661.2/A1Z1Q3)

MGSSHHHHHHSSGLVPRGSHMENLYFQVWREEKERLLKMTLEERRKEYLRDYIPLNSILSWKEEMKGKGQNDEENTQ
ETSQVKKSLTEKVSLYRGDITLLEVDAIVNAANASLLGGGGVDGCIHRAAGPCLLAECRNLNGCDTGHAKITCGYDL
PAKYVIHTVGPIARGHINGSHKEDLANCYKSSLKLVKENNIRSVAFPCISTGIYGFPNEPAAVIALNTIKEWLAKNH
HEVDRIIFCVFLEVDFKIYKKKMNEFFSV*

atgggcagcagccatcatcatcatcatcacagcagcggcctggtgccgcgcggcagccatatggagaacctgtactt
ccaatccGTGTGGAGAGAGGAGAAAGAACGTTTATTGAAGATGACCTTAGAAGAGAGACGCAAAGAATACCTAAGAG
ACTATATTCCCCTGAACAGCATTCTATCATGGAAGGAGGAGATGAAGGGCAAGGGCCAAAATGATGAAGAAAATACT
CAGGAAACATCCCAGGTGAAGAAAAGTTTGACTGAAAAAGTTTCTCTCTATAGAGGTGACATCACATTGCTAGAGGT
AGATGCTATAGTCAATGCCGCAAATGCCAGTCTTCTTGGAGGAGGAGGTGTGGATGGCTGTATTCATAGAGCAGCCG
GCCCCTGTTTGCTAGCTGAATGTCGTAACCTGAATGGCTGTGATACTGGACATGCAAAAATCACATGTGGCTATGAC
CTTCCTGCAAAATATGTCATCCATACTGTAGGGCCAATAGCCAGGGGCCATATTAATGGTTCCCACAAGGAAGACCT
TGCAAATTGCTATAAATCATCTCTGAAGCTCGTGAAAGAAAATAACATCCGATCAGTTGCATTTCCCTGCATCTCAA
CAGGCATTTATGGCTTTCCCAACGAGCCTGCTGCAGTCATTGCCCTCAACACCATTAAGGAATGGCTTGCCAAGAAT
CACCATGAGGTGGATCGGATCATTTTCTGTGTCTTCTTAGAAGTTGACTTCAAAATCTACAAAAAGAAAATGAATGA
GTTTTTCTCCGTATGATGA

SARS-CoV-2 Macro1 (aa1024-1192) (YP_009724389.1/P0DTD1)

MGSDKIHHHHHHSSGENLYFQGEVNSFSGYLKLTDNVYIKNADIVEEAKKVKPTVVVNAANVYLKHGGGVAGALNKA
TNNAMQVESDDYIATNGPLKVGGSCVLSGHNLAKHCLHVVGPNVNKGEDIQLLKSAYENFNQHEVLLAPLLSAGIFG
ADPIHSLRVCVDTVRTNVYLAVFDKNLYDKLVSSFLE*

CCATGGGTAGCGACAAGATCCACCACCACCACCACCACAGCAGCGGCGAGAACCTGTACTTCCAGGGTGAAGTGAAC
AGCTTTAGCGGCTACCTGAAGCTGACCGACAACGTTTATATCAAAAACGCGGATATTGTGGAGGAAGCGAAGAAAGT

```
TAAACCGACCGTGGTTGTGAACGCGGCGAACGTGTATCTGAAGCATGGTGGCGGTGTTGCGGGTGCGCTGAACAAAG
CGACCAACAACGCGATGCAAGTGGAGAGCGACGATTATATTGCGACCAACGGTCCGCTGAAAGTGGGCGGTAGCTGC
GTTCTGAGCGGCCACAACCTGGCGAAACACTGCCTGCACGTTGTGGGCCCGAACGTGAACAAGGGTGAAGATATTCA
GCTGCTGAAAAGCGCGTATGAGAACTTCAACCAACACGAAGTGCTGCTGGCGCCGCTGCTGAGCGCGGGTATCTTTG
GTGCGGACCCGATTCACAGCCTGCGTGTTTGCGTGGATACCGTTCGTACCAACGTGTACCTGGCGGTTTTCGACAAG
AACCTGTATGATAAACTGGTTAGCAGCTTTCTGGAGTAA
```

General Synthetic Experimental Details

Unless otherwise stated, all reactions were carried out under air atmosphere in reaction flasks. Commercially available reagents and solvents from Sigma Aldrich, TCI, Alfa-aesar, Combi-blocks, Chem-impex international, Ambeed, VWR, and Fisher scientific, USA were used as received. All isolated compounds were characterized by ^{1}H NMR and ^{13}C NMR spectra and recorded on Bruker 400MHz, and 500MHz spectrometers at Department of Chemistry and Chemical Biology, Cornell University. Chemical shifts (δ) are reported in ppm reference to the solvent peaks of $CDCl_3$ (7.24 ppm) for ^{1}H NMR and 77.0(+/- 0.77) for ^{13}C NMR respectively. The following abbreviations were used to indicate multiplicity: s (singlet), bs (broad singlet), d (doublet), t (triplet), q (quartet), dd (double doublets), td (triplet of doublet), and m (multiplet). The mass spectrometry data were obtained on a ThermoFisher Scientific Exactive series DART Mass Spectrometer using LCMS. For column chromatography, silica gel (400–200 mesh) from VWR was used. A gradient elution using petroleum-ether/dichloromethane and ethyl acetate/ methanol was performed, based on Merck aluminum-supported TLC sheets (silica gel 60F254). For reverse phase C18 column chromatography, water and acetonitrile were used as the solvents on a Shimazu HPLC.

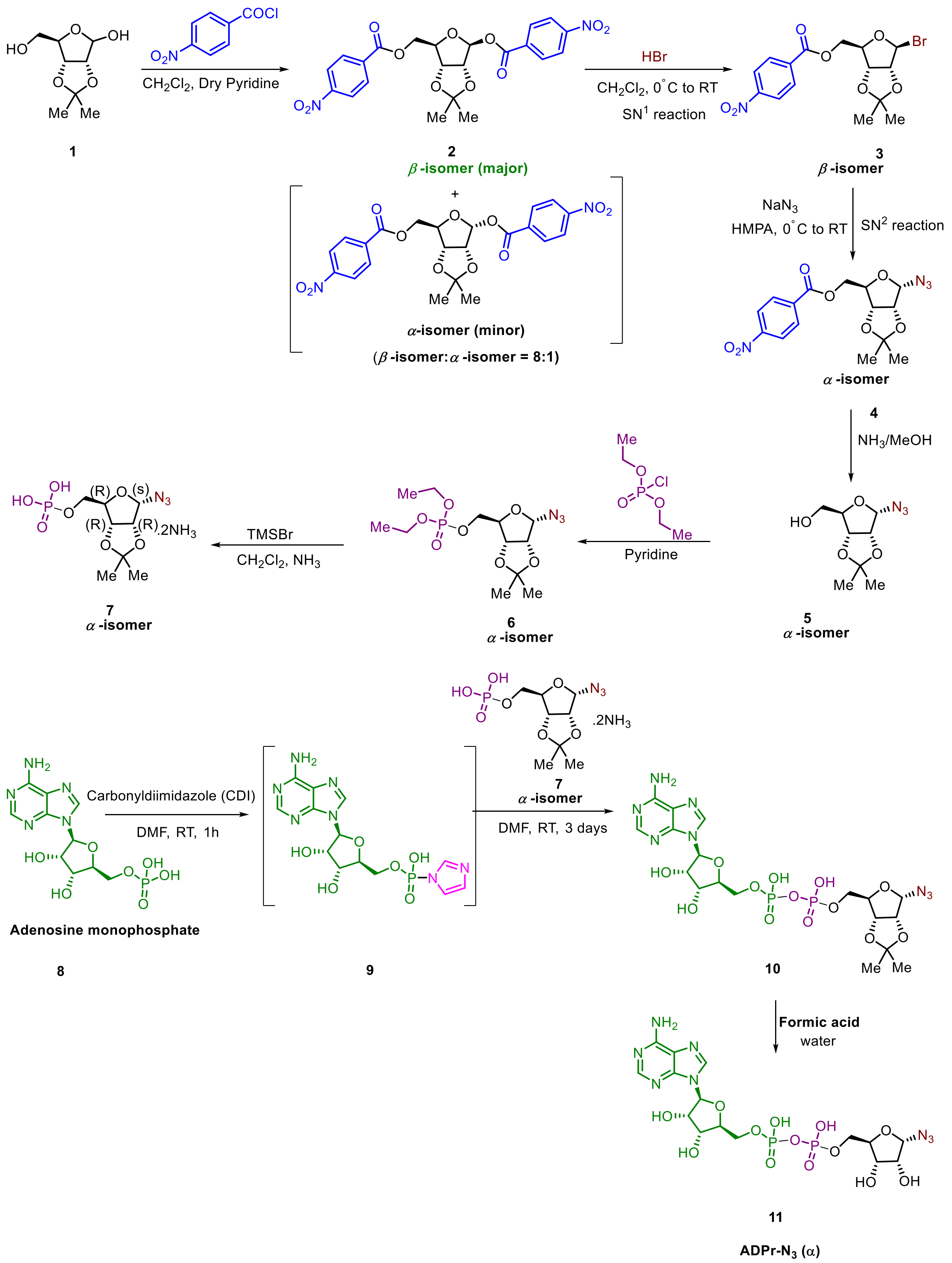

Scheme 1: Synthesis of ADP-Ribose-N3(α)

Synthesis of ADP-Ribose-N_3(α)

Synthesis of 2,3-O-isopropylidene-1,5-di-O-*p*-nitrobenzoyl-D-ribose (compound 2). 2,3-O-Isopropylidene-D-ribofuranose (compound **1**, 4.00 g, 21.0 mmol) was dissolved in dry pyridine (50 mL) and cooled to 0 °C. To this solution was added 4-nitrobenzoyl chloride (9.84 g, 53.0 mmol) slowly in portions. The reaction was stirred at 0 °C for 1 hour and then at room temperature for 20 hours. After completion of reaction, TLC showed mixture of β and α (8:1). Then, solvent was removed, and the residue was dissolved in dichloromethane (DCM) and washed with water, 0.1 N HCl, and brine. The combined organic layer was dried over Na_2SO_4 and concentrated using a rotavapor. The reaction mixture was purified by silica gel column chromatography using petroleum ether/ethyl acetate (3:1, v:v) as the eluent and offered the desired produc**t 2** as major *β*-isomer as a colorless solid (9.75 g, 95% yield). ^{1}H NMR (500 MHz, $CDCl_3$) δ 8.24 (dd, J = 8.7, 6.8 Hz, 4H), 8.17 – 8.11 (m, 4H), 6.51 (s, 1H, anomeric), 5.00 (d, J = 5.9 Hz, 1H), 4.93 (d, J = 5.7 Hz, 1H), 4.75 (t, J = 7.0 Hz, 1H), 4.56 – 4.45 (m, 2H), 1.56 (s, 3H), 1.39 (s, 3H). ^{13}C NMR (126 MHz, $CDCl_3$) δ 164.13, 163.13, 150.79, 150.71, 134.67, 130.84, 130.81, 123.68, 123.59, 113.74, 103.88, 85.62, 85.24, 81.36, 65.15, 26.42, 25.00. HRMS (m/z): [$M+NH_4^+$] calcd for $C_{22}H_{24}N_3O_{11}^+$: 506.1405; found, 506.1399. TLC: R_f = 0.5 (50:50 petroleum ether:EtOAc).

Synthesis of 2,3-O-isopropylidene-5-O-p-nitrobenzoyl-D-ribofuranosyl bromide (compound 3). PPh_3HBr (34.6 g) was suspended in xylene (70 mL) and heated to refluxing. Gas developed was transferred out to and absorbed by cooled DCM (50 mL). After 1 hour, the cold DCM solution was weighed (2.08 g of HBr, 0.51 mmol) and used in the next step reaction directly.

To a solution of HBr in DCM (50 mL, 0.51 mmol) at 0 °C was added compound **2** (3.00 g, 6.14 mmol). The reaction flask was sealed and stirred for 30 min and then warmed up to room temperature for 1 hour. The reaction was filtered to remove solid. The filtrate was concentrated and the residue was dissolved in ether (30 mL) and hexane (20 mL) and concentrated to get solid product **3** as a brown solid (2.23 g, 90% yield). This step occurs as an SN1 reaction conditions to form **compound 3** as β-isomer**.**^{1}H NMR (500 MHz, $CDCl_3$) δ 8.31 (d, J = 6.9 Hz, 2H), 8.27 (d, J = 8.9 Hz, 2H), 6.50 (s, 1H, anomeric), 5.29 (dd, J = 5.8, 1.7 Hz, 1H), 4.99 (d, J = 5.6 Hz, 1H), 4.71 (qd, J = 4.1, 1.6 Hz, 3H), 1.51 (s, 3H), 1.37 (s, 3H). ^{13}C NMR (126 MHz, $CDCl_3$) δ 164.21, 150.75, 134.85, 131.00, 123.63, 114.16, 91.60, 90.29, 87.97, 81.15, 63.53, 26.67, 25.44. HRMS (m/z): [$M+NH_4$] calcd for C15H20BrN$_2$O7: 419.0454; found, 419.0432 TLC: R_f = 0.45 (90:10 petroleum ether:EtOAc).

Synthesis of 2,3-O-isopropylidene-5-O-(p-nitrobenzoyl)-α-D-ribofuranosyl azide (compound 4). To a cold solution of NaN_3 (1.94 g, 29.8 mmol) in HMPA (20 mL) at 0 °C was added compound **3** (4.00 g, 9.95 mmol). The resulting solution was stirred at 0 °C for 1 hour. The reaction was monitored by TLC. If starting material remained, additional 0.0 g of NaN_3 was added and stirred until starting material was consumed. The reaction mixture was poured into water and extracted with 50% ether/ethyl acetate (v/v) four times. The combined organic solution was washed with water, brine and dried over anhydrous Na_2SO_4. After concentration, the residue (α:β ~50:1) was recrystallized in t-butyl methyl ether to afford α-**compound 4**. The mother liquid was purified by silica gel column chromatography (DCM: Ether: Hexane= 1:4:10), This led to the elution of first the β isomer and then another crop of α isomer of compound **4.** This step is crucial

for the transformation of β to α isomer in Scheme 1. Azido bond formation from bromo derivatives proceed through SN2 because azide ion (N_3^-) is an excellent nucleophile**.** Yield of **4**: (1.63 g, 45%) ^{1}H NMR (500 MHz, $CDCl_3$) δ 8.35 – 8.31 (m, 2H), 8.23 – 8.18 (m, 2H), 5.14 (d, J = 4.2 Hz, 1H, anomeric), 4.82 (dd, J = 6.5, 4.3 Hz, 1H), 4.76 (dd, J = 6.5, 2.2 Hz, 1H), 4.59 (td, J = 4.7, 2.3 Hz, 1H), 4.56 – 4.46 (m, 2H), 1.64 (s, 3H), 1.40 (s, 3H).^{13}C NMR (126 MHz, $CDCl_3$) δ 164.25, 150.80, 134.72, 130.81, 123.76, 115.32, 90.66, 81.29, 81.23, 80.54, 65.15, 25.76, 25.01. HRMS (m/z): [$M+NH_4^+$] calcd for $C_{15}H_{20}N_5O_7^+$: 382.1357; found, 382.1327. TLC: R_f = 0.45 dichloromethane/diethyl ether/petroleum ether (1:4:10, v/v/v).

Synthesis of 2,3-O-Isopropylidene-α-D-ribofuranosyl azide (compound 5). To a cold solution of NH_3 in methanol (7 M, 5 mL) at 0 °C was added to compound **4**-α (100 mg, 0.28 mmol). The reaction mixture was stirred for 12 hours in a cold room (4 °C). Solvent was evaporated and the yellowish-brown solid residue was purified by column chromatography on silica gel using petroleum ether/ethyl acetate (80:20, v/v) as eluent to afford the desired compound **5** as a yellow oil (57.0 mg, 97%). ^{1}H NMR (500 MHz, CDCl3) δ 5.15 (d, J = 4.0 Hz, 1H, anomeric), 4.77 – 4.72 (m, 2H), 4.30 (td, J = 3.6, 1.8 Hz, 1H), 3.82 (dd, J = 11.8, 3.1 Hz, 1H), 3.70 (dd, J = 11.8, 3.9 Hz, 1H), 2.42 – 2.17 (m, 1H), 1.60 (s, 3H), 1.37 (s, 3H). ^{13}C NMR (126 MHz, CDCl3) δ 114.71, 90.96, 83.31, 81.47, 81.09, 63.22, 25.67, 24.87. HRMS (m/z): [$M+NH_4+$] calcd for $C_8H_{17}N_4O_4^+$: 233.1244; found, 233.1227**.** TLC: R_f = 0.45 (60:40 petroleum ether:EtOAc).

Synthesis of 2,3-O-Isopropylidene-5-O-(diethyl phosphonate)-α-D-ribofuranosyl azide (compound 6). Diethyl phosphorochloridate (138 µL, 0.95 mmol) was added to a solution of compound **5** (57 mg, 0.26 mmol) in 5 mL of pyridine at 0 °C. The reaction mixture was stirrer at 0 °C for 2 hours and monitored by TLC. After completion, the reaction was quenched by 3 mL of methanol. The solvent was removed using a rotavapor, and the residue was redissolved in ethyl acetate and washed with water and brine. The combined organic layer was dried over anhydrous Na_2SO_4 and concentrated. The residue was purified by silica gel column chromatography using hexane/ethyl acetate (50:50, v/v) to provide the desired product **6** as a yellow oil (107 mg, 80% yield). ^{1}H NMR (500 MHz, CDCl3) δ 5.02 (d, J = 4.2 Hz, 1H, anomeric), 4.74 (dd, J = 6.4, 1.7 Hz, 1H), 4.69 (dd, J = 6.4, 4.2 Hz, 1H), 4.33 (dt, J = 4.6, 2.3 Hz, 1H), 4.11 – 4.05 (m, 6H), 1.54 (s, 3H), 1.32 – 1.28 (m, 9H). ^{13}C NMR (126 MHz, CDCl3) δ 114.41, 90.99, 81.27, 81.21, 81.10, 67.86, 64.12, 64.08, 25.56, 24.70, 16.10, 16.05. HRMS (m/z): [$M+H^+$] calcd for $C_{12}H_{23}N_3O_7P^+$: 352.1268; found, 352.1269. TLC: R_f = 0.45 (30:70 Hexane:EtOAc).

Synthesis of 2,3-O-Isopropylidene -5- O-(dihydrogen phosphonate)- α-D-ribofuranosyl azide (compound 7). Trimethylbromosilane (TMSBr, 178 µL, 1.34 mmol) was added to a solution of compound **6** (0.22 mmol, 79 mg) in dichloromethane (6 mL). The reaction mixture was stirrer at room temperature for 24 hours. After completion, the solvent was evaporated, and the residue was redissolved in dioxane (5 mL) and 2 mL of 7M NH_3/MeOH solution and stirrer for 30 min at room temperature. The solvents were removed using a rotavapor, and the residue was completely dried under high vacuum. The solid was suspended in 10 mL of ether and stirrer for 2-3 h. The solution was filtered to afford compound **7** as colorless solid (59.1 mg, 73% yield)**.** ^{1}H NMR (400 MHz, $CDCl_3$) δ 5.04 (s, 1H, anomeric), 4.81 – 4.65 (m, 2H), 4.30 (s, 1H), 3.85 (s, 2H), 3.64 (s, 1H), 3.42 (q, 1H), 1.50 (s, 3H), 1.31 (s, 3H). ^{13}C NMR (126 MHz, DMSO) δ 112.78, 90.28, 81.92,

81.28, 65.30, 31.17, 25.73, 24.73. ^{31}P NMR (202 MHz, DMSO) δ -0.73, -1.50. LCMS (ESI): [$M+NH_4^+$] calcd for $C_8H_{18}N_4O_7P^+$: 313.09; found, 313.1.

Synthesis of compound 10. CDI (0.15 mmol, 24.6 mg) was added to a solution of AMP (0.15 mmol, 55 mg) in 5 mL of DMF. The solution was stirrer at room temperature for 2 hours. LCMS showed that AMP was completely converted to **9**. Compound **7** (50 mg, 0.15 mmol) was added to the reaction and the solution was stirred at room temperature for 2 days. LCMS was used to check that **9** was completely consumed. Next, DMF was removed by lyophilized and residue was purified by HPLC using water with 0.1% TFA as solvent A and acetonitrile with 0.1% TFA as solvent B. The flow rate was 10 mL/min. The solvent gradient program was the following: 0% solvent B for the first 3 min, followed by a liner progression to 6% solvent B for next 30 minutes, then 5 minutes of 80% solvent B and 5 minutes of 100% solvent A for a total of 43 minutes per HPLC run. The product peak came out at 9 min (~1.65% of solvent B) and lyophilization of the collected fraction yielded colorless solid **10** (55.4 mg, 56% yield). ^1H NMR (500 MHz, D_2O) δ 8.65 (d, J = 2.9 Hz, 1H), 8.43 (s, 1H), 7.46 (s, 2H), 6.18 (d, J = 5.4 Hz, 1H), 5.51 (d, J = 4.7 Hz, 1H), 5.43 (d, J = 4.7 Hz, 1H, anomeric), 5.38 (d, J = 4.3 Hz, 1H), 4.98 (d, J = 6.2 Hz, 1H), 4.94 (s, 1H), 4.54 (t, J = 4.3 Hz, 1H), 4.44 – 4.39 (m, 2H), 4.29 (d, J = 5.2 Hz, 2H), 4.21 – 4.19 (m, 2H), 4.04 (d, J = 4.4 Hz, 1H), 3.97 (d, J = 5.2 Hz, 2H), 1.57 (s, 3H), 1.40 (s, 3H). ^{13}C NMR (126 MHz, D_2O) δ 163.18, 162.89, 144.81, 142.53, 133.41, 117.53, 115.21, 91.48, 87.98, 84.10, 81.42, 80.39, 74.67, 71.63, 69.72, 64.70, 24.57, 23.57. ^{31}P NMR (202 MHz, D_2O) δ 0.32, -11.26. LCMS (m/z): [$M+H^+$] calcd for $C_{18}H_{27}N_8O_{13}P_2^+$: 625.1; found, 625.2.

Synthesis of compound 11. Compound **10** (50 mg, 0.08 mmol) was dissolved in 2 mL of water and 2 mL of 88% of formic acid was added to this solution at room temperature. The solution was stirred at room temperature overnight. The solvent was removed by lyophilization and the residue was purified by HPLC using water with 0.1% TFA as solvent A and acetonitrile with 0.1% TFA as solvent B. The flow rate was 10 mL/min. The solvent gradient program was the following: 0% solvent B for the first 3 min, followed by a liner progression to 12% solvent B in next 30 minutes, then to 30% solvent B for in the next 17 min, then end with 5 minutes of 80% solvent B and 5 minutes of 100% solvent A for a total of 60 minutes per HPLC run. The product peak came out at 9 min (~ 2.5% of solvent B) and lyophilization of the collected fraction yielded **11** as a colorless solid (29.5 mg, 63% yield).[1-3] The stereochemistry of ADP-N_3 was determined by comparing with a previous report, with α-C-1 proton at δ 5.44 ppm and β-C-1 proton at δ 5.33 ppm.[2] ^1H NMR (300 MHz, D_2O) (*α*-isomer) δ 8.44 (s, 1H), 8.23 (s, 1H), 7.25 (s, 2H), 5.98 (d, J = 5.2 Hz, 1H), 5.44 (d, *J* = 4.5 Hz,1H, anomeric), 4.37 – 4.30 (m, 1H), 4.20 (s, 1H), 4.10 – 4.04 (m, 4H), 4.03 – 3.97 (m, 2H), 3.89 – 3.82 (m, 2H). ^1H NMR (300 MHz, D_2O) (*β*-isomer) δ 8.44 (s, 1H), 8.23 (s, 1H), 7.25 (s, 2H), 5.98 (d, J = 5.2 Hz, 1H), 5.33 – 5.21 (m, 1H), 4.37 – 4.30 (m, 1H), 4.20 (s, 1H), 4.10 – 4.04 (m, 4H), 4.03 – 3.97 (m, 2H), 3.89 – 3.82 (m, 2H). ^{13}C NMR (126 MHz, D_2O) δ 162.84, 149.95, 148.43, 144.70, 142.56, 117.50, 115.18, 91.45, 88.03, 83.83, 74.66, 71.62, 70.31, 69.70, 65.55, 65.22. ^{31}P NMR (202 MHz, D_2O) δ 0.15, -11.24. HRMS (m/z): [$M+H^+$] calcd for $C_{15}H_{23}N_8O_{13}P_2^+$: 585.08; found, 585.08. Purity: ~85%.

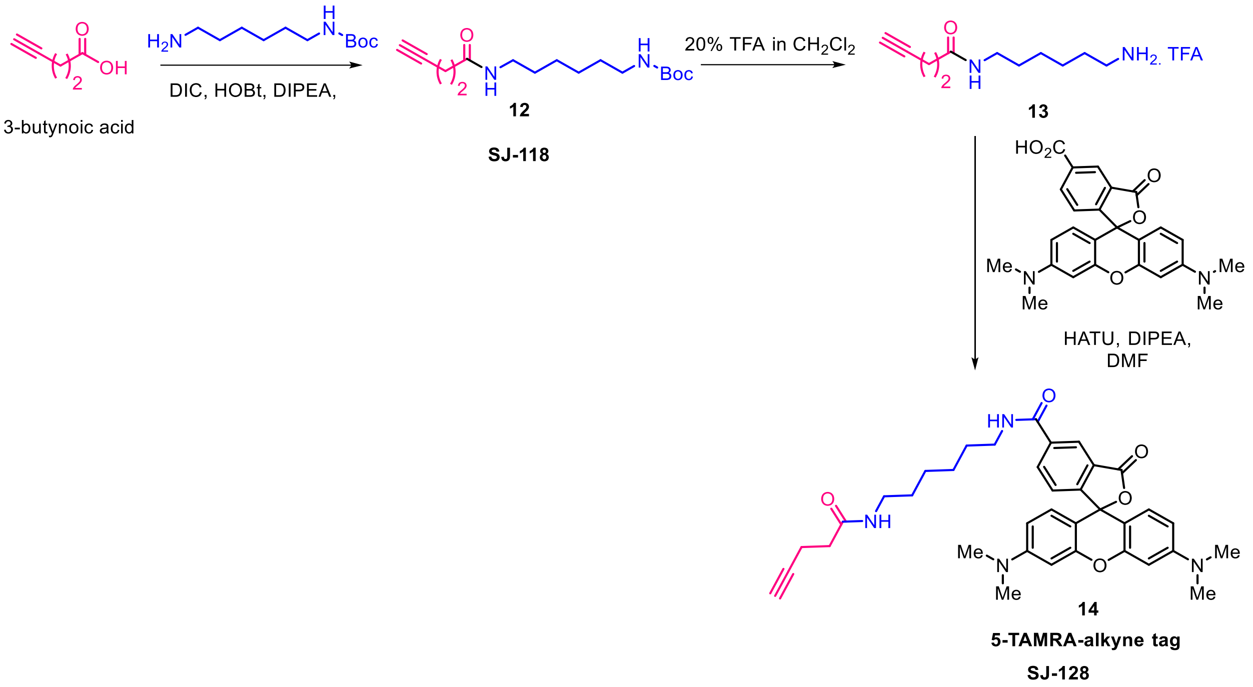

Scheme 2: Synthesis of 5-TAMRA-alkyne tag

Synthesis of tert-butyl (6-(pent-4-ynamido)hexyl)carbamate (compound 12).[4] 4-pentynoic acid (1.02 mmol, 100 mg), 1-hydroxybenzotriazole hydrate (HOBT) (0.10 mmol, 14 mg), and *N*-(3-dimethylaminopropyl)-N′-ethylcarbodiimide hydrochloride (EDC-HCl, 1.22 mmol, 234 mg) were dissolved in 5 mL dichloromethane and stirrer for min in room temperature. To this reaction mixture, *N*-Boc-1,6-hexanediamine (1.12 mmol, 243 mg) was added and the reaction was stirred overnight. The solvent was evaporated, and the crude residue was purified by silica gel column chromatography using hexane/ethyl acetate (90:10, v/v) as the solvent. Compound 12 was obtained as a colorless solid (218 mg, 72% yield). ^{1}H NMR (500 MHz, $CDCl_3$) δ 5.97 (s, 1H), 4.61 (s, 1H), 3.26 (q, J = 6.7 Hz, 2H), 3.11 (q, J = 6.7 Hz, 2H), 2.53 (td, J = 7.2, 2.6 Hz, 2H), 2.40 (t, J = 7.2 Hz, 2H), 2.01 (d, J = 5.3 Hz, 1H), 1.54 – 1.46 (m, 4H), 1.44 (s, 9H), 1.34 (dq, J = 8.5, 4.7 Hz, 4H). ^{13}C NMR (126 MHz, $CDCl_3$) δ 170.94, 156.12, 83.11, 79.08, 69.25, 40.17, 39.22, 35.39, 29.98, 29.39, 28.43, 26.16, 26.04, 14.97. HRMS (m/z): [M+H^{+}] calcd for $C_{16}H_{29}N_2O_3^{+}$: 297.2173; found, 267.3152. TLC: R_f = 0.45 (80:20 Hexane:EtOAc).

Synthesis of 3',6'-bis(dimethylamino)-3-oxo-N-(6-(pent-4-ynamido)hexyl)-3H-spiro[isobenzofuran-1,9'-xanthene]-5-carboxamide (compound 14).[5] Compound **12** (50.0 mg, 0.169 mmol) was dissolved in 4 mL of dichloromethane and 1 mL of trifluoroacetic was added to this solution (20% TFA in DCM). The reaction mixture was stirred at room temperature for 2 hours. The crude reaction was check using LCMS to confirm that the desired product **13** was formed and then the solvent was removed using a rotavapor. The residue was dissolved in methanol and evaporated using a rotavapor. The process was repeated by 6-7 times. The residue was dried

under high vacuum overnight and the crude **13** was used in the next step without further purification.

5-carboxyteramethylrhodamine (5-TAMRA) (43 mg, 0.10 mmol) was dissolved in 2 mL DMF and mixed with HATU (46.0 mg, 0.12 mmol). The reaction mixture was stirrer for 10 min to ensure that it was fully dissolved. Compound **13**-TFA salt (38 mg, 0.12 mmol) was then added, followed by 54.0 µL of N,N-diisopropylethylamine (0.30 mmol). The reaction mixture was stirred for 3 h and was concentrated upon completion as determined by TLC. The crude reaction mixture was purified by HPLC using water with 0.1% TFA as solvent A and acetonitrile with 0.1% TFA as solvent B. The flow rate was 10 mL/min. The solvent gradient program was the following: starting with 20.0% solvent B, followed by a liner progression to 25.5% solvent B for the next 20 minutes, end with 10 minutes of 80% solvent B and then 10 minutes of 100% solvent A for a total of 40 minutes per HPLC run. The product peak came out at 15 min, and lyophilization of the collected fraction yielded compound 14 as a pink solid (31.3 mg, 42% yield). ^{1}H NMR (500 MHz, CD_3CN) δ 8.65 (s, 1H), 8.21 (d, J = 7.9 Hz, 1H), 7.66 (s, 1H), 7.39 (d, J = 7.9 Hz, 1H), 7.05 (d, J = 9.3 Hz, 2H), 6.86 (d, J = 8.0 Hz, 2H), 6.79 (d, J = 2.4 Hz, 2H), 6.56 (s, 1H), 3.43 (d, J = 6.5 Hz, 2H), 3.22 (s, 12H), 3.18 (d, J = 7.0 Hz, 2H), 2.45 – 2.43 (m, 2H), 2.34 – 2.32 (m, 2H), 2.20 – 2.19 (m, 1H), 1.64 (t, J = 7.1 Hz, 1H), 1.52 – 1.47 (m, 1H), 1.40 (d, J = 11.1 Hz, 3H), 1.30 (d, J = 5.9 Hz, 3H). ^{13}C NMR (126 MHz, CD3CN) δ 170.77, 167.65, 167.00, 165.44, 156.62, 156.52, 146.16, 136.54, 131.37, 130.54, 129.78, 129.05, 128.45, 113.31, 112.10, 96.50, 83.49, 69.04, 40.16, 39.48, 38.63, 34.68, 31.65, 29.16, 29.03, 26.08, 25.95. LCMS (ESI): [M+H] calcd for $C_{36}H_{41}N_4O_5^+$: 609.3; found, 609.3.

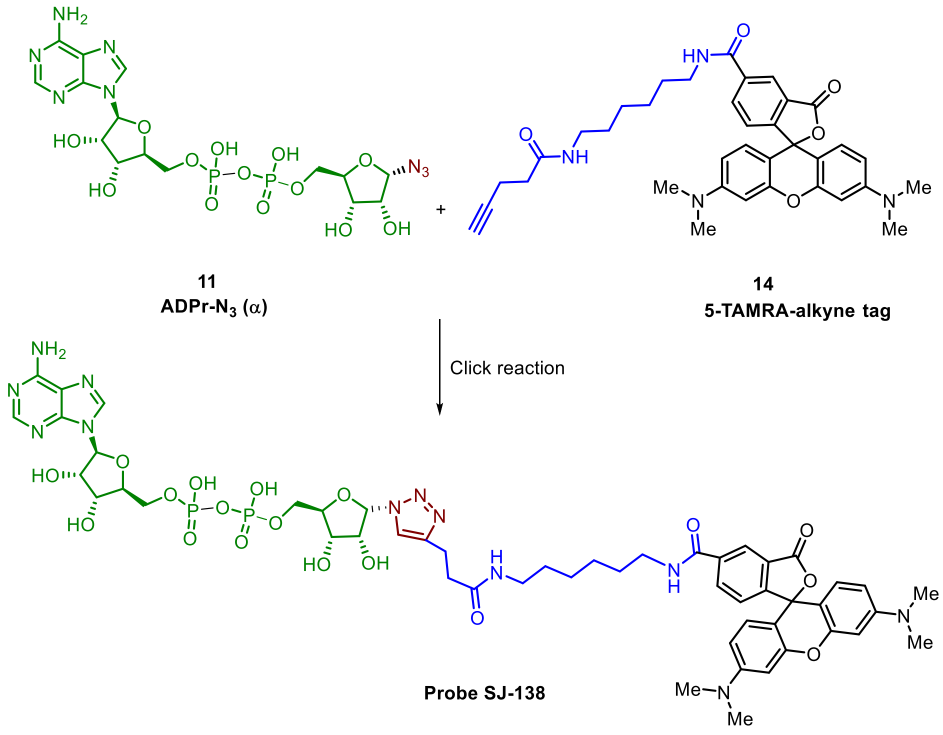

Scheme 3: Synthesis of ADP-ribosylated Fluorescence Probe SJ-138 (**TAMRA-ADPr**)

Synthesis of TAMRA-ADPr.[2, 6] α-ADP-ribosyl-N_3 (**11**) (3 eq., 0.030 mmol, 20 mg), compound **14** (1 eq., 0.01 mmol, 6.8 mg), 20 mol% $CuSO_4 \cdot 5H_2O$ (0.2 equiv., 0.002 mmol, 0.6 mg), 20 mol% Cu(0) (copper powder, 0.002 mmol, 0.2 mg), sodium ascorbate (0.6 eq., 0.0067 mmol, 1.33 mg) and $NaHCO_3$ (4.5 eq., 0.05 mmol, 4.22 mg) were added to water:*t*-BuOH (1:1) mixture (1 mL) and stirred for 24 h at room temperature. The reaction was monitored by LCMS. After the reaction was completed, the product was purified using HPLC. The HPLC used water with 0.1% TFA as solvent A and acetonitrile with 0.1% TFA as solvent B. The flow rate was 10 mL/min. The solvent gradient program was the following: starting with 0% solvent B f, followed by a liner progression to 12% solvent B for the next 30 minutes, then progressing to 30% solvent B for in the next 20 min, ending with t 5 minutes of 80% solvent B and then 5 minutes of 100% solvent A for a total of 60 minutes per HPLC run. The product peak came out at 38 min (~19% of solvent B) and lyophilization of the collected fraction yielded TAMRA-ADPr as a pink solid (5.5 mg, 41%). ^{1}H NMR (500 MHz, DMSO) δ 8.74 (d, *J* = 4.4 Hz, 1H), 8.50 (d, *J* = 8.4 Hz, 2H), 8.25 – 8.15 (m, 1H), 7.50 (dd, *J* = 8.4, 4.3 Hz, 1H), 7.43 – 7.27 (m, 1H), 6.65 (d, *J* =8.0 Hz, 1H), 5.94 (s, 1H), 4.93 (s, 1H, (anomeric proton), 4.54 (s, 1H), 4.28 (s, 1H), 4.02 (s, 2H), 3.58 (s, 12H), 3.10 (q, *J* = 7.3 Hz, 4H), 3.03 (d, *J* = 6.8 Hz, 3H), 2.88 (s, 3H), 2.71 (d, J = 2.9 Hz, 1H), 2.59 (s, 1H), 2.36 – 2.31 (m, 1H), 2.25 (dt, J = 9.8, 4.9 Hz, 1H), 1.56 – 1.48 (m, 1H), 1.38 (q, *J* = 6.5 Hz, 1H). ^{13}C NMR (126 MHz, DMSO) δ 173.40, 170.79, 169.12, 165.25, 161.28, 158.76, 158.51, 152.99, 151.49, 140.05, 135.09, 129.50, 129.22, 121.24, 87.36, 84.23, 75.86, 74.33, 71.67, 44.69, 42.21, 40.43, 40.29, 40.20, 40.12, 40.03, 39.95, 39.87, 39.79, 39.70, 39.62, 39.53, 39.36, 39.20, 38.80, 38.68, 34.60, 29.55, 29.43, 29.33, 26.60, 26.50, 26.29, 26.16, 25.63, 18.42, 17.07, 14.74, 12.78. ^{31}P NMR (202 MHz, DMSO) δ -11.65. LCMS (ESI): [M+H] calcd for $C_{51}H_{63}N_{12}O_{18}P_2^+$: 1193.4; found, 1193.4.

References:

1. Li, L.; Li, Q.; Ding, S.; Xin, P.; Zhang, Y.; Huang, S.; Zhang, G. ADP-ribosyl-N3: A Versatile Precursor for Divergent Syntheses of ADP-ribosylated Compounds. Molecules 2017, 22, 1346. https://doi.org/10.3390/molecules22081346
2. Zhu, A.; Li, X.; Bai, L.; Zhu, G.; Guo, Y.; Lin, J.; Cui, Y.; Tian, G.; Zhang, L.; Wang, J.; Li, X. D.; Li, L., Biomimetic α-selective ribosylation enables two-step modular synthesis of biologically important ADP-ribosylated peptides. Nat. Commun. 2020, 11 (1), 5600.
3. Minnee, H.; Rack, J. G. M.; van der Marel, G. A.; Overkleeft, H. S.; Codée, J. D. C.; Ahel, I.; Filippov, D. V., Mimetics of ADP-Ribosylated Histidine through Copper(I)-Catalyzed Click Chemistry. Organic Letters 2022, 24 (21), 3776-3780.
4. Mishra, N. M.; Briers, Y.; Lamberigts, C.; Steenackers, H.; Robijns, S.; Landuyt, B.; Vanderleyden, J.; Schoofs, L.; Lavigne, R.; Luyten, W.; Van der Eycken, E. V., Evaluation of the antibacterial and antibiofilm activities of novel CRAMP–vancomycin conjugates with diverse linkers. Organic & Biomolecular Chemistry 2015, 13 (27), 7477-7486.
5. PCT/US2021/017839/Linking amino acid sequences, manufacturing method thereof, and use thereof/ WO/2021/163467/, 08/19/2021

6. Jiang, H.; Congleton, J.; Liu, Q.; Merchant, P.; Malavasi, F.; Lee, H. C.; Hao, Q.; Yen, A.; Lin, H., Mechanism-Based Small Molecule Probes for Labeling CD38 on Live Cells. *J. Am. Chem. Soc*. 2009, 131 (5), 1658-1659.

^{1}H NMR: compound **2**

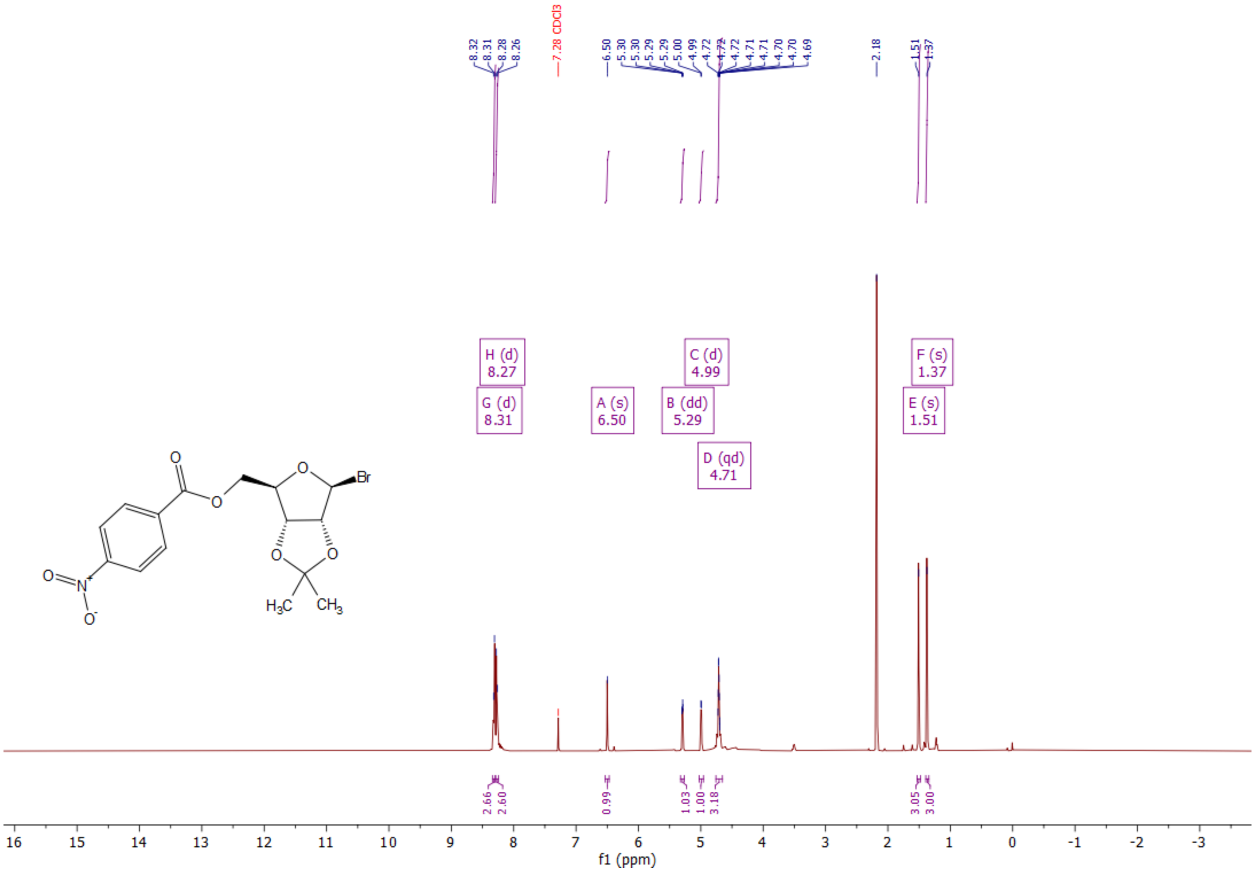

^{13}C NMR compound **2**

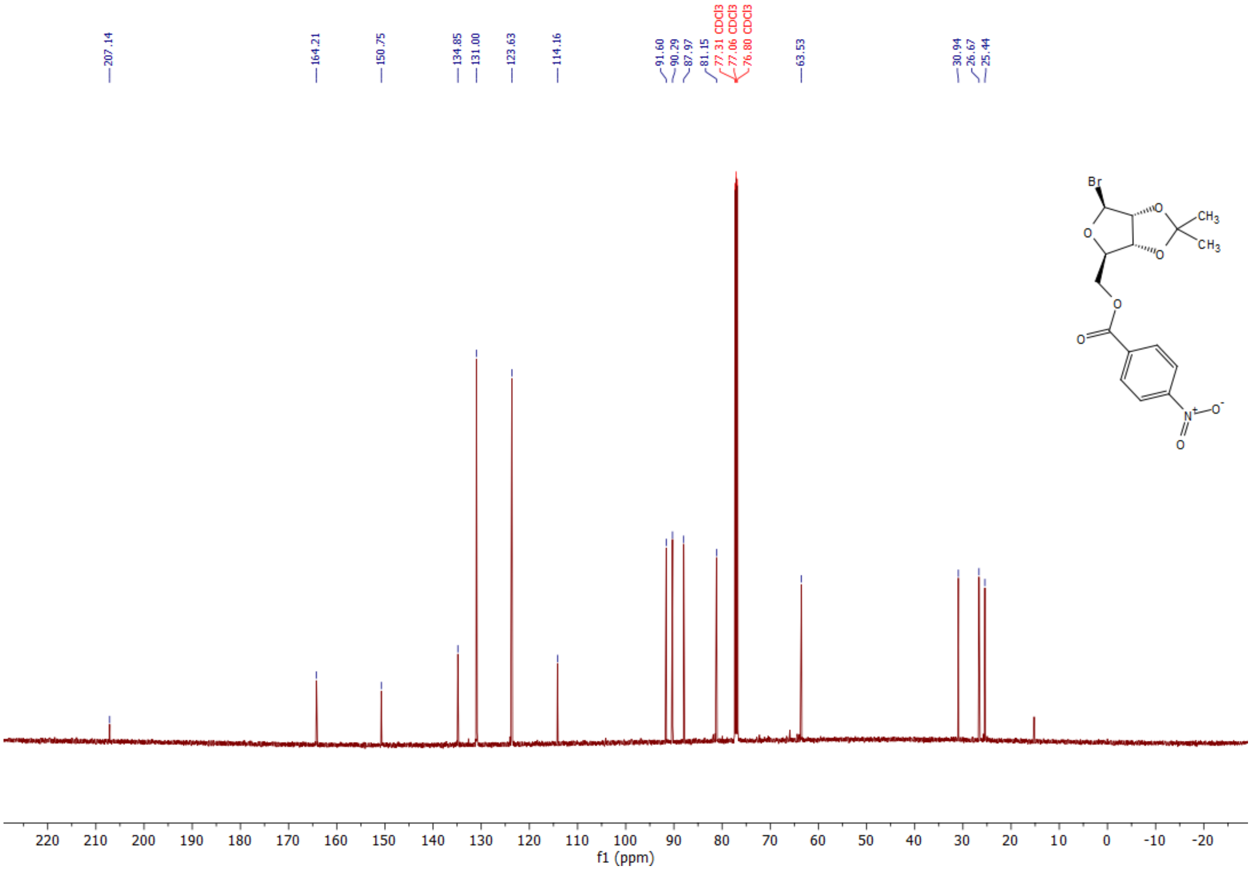

^{1}H NMR compound **3**

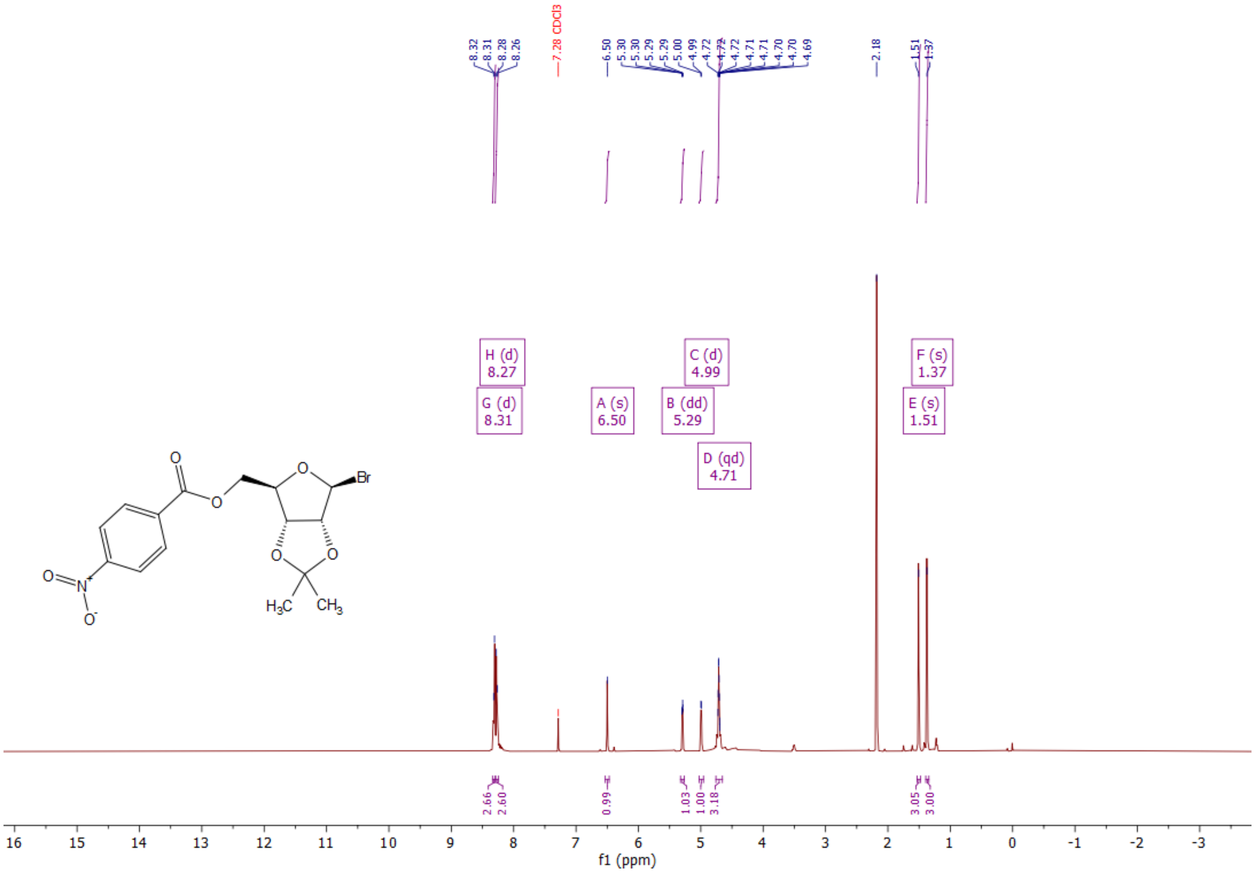

^{13}C NMR compound **3**

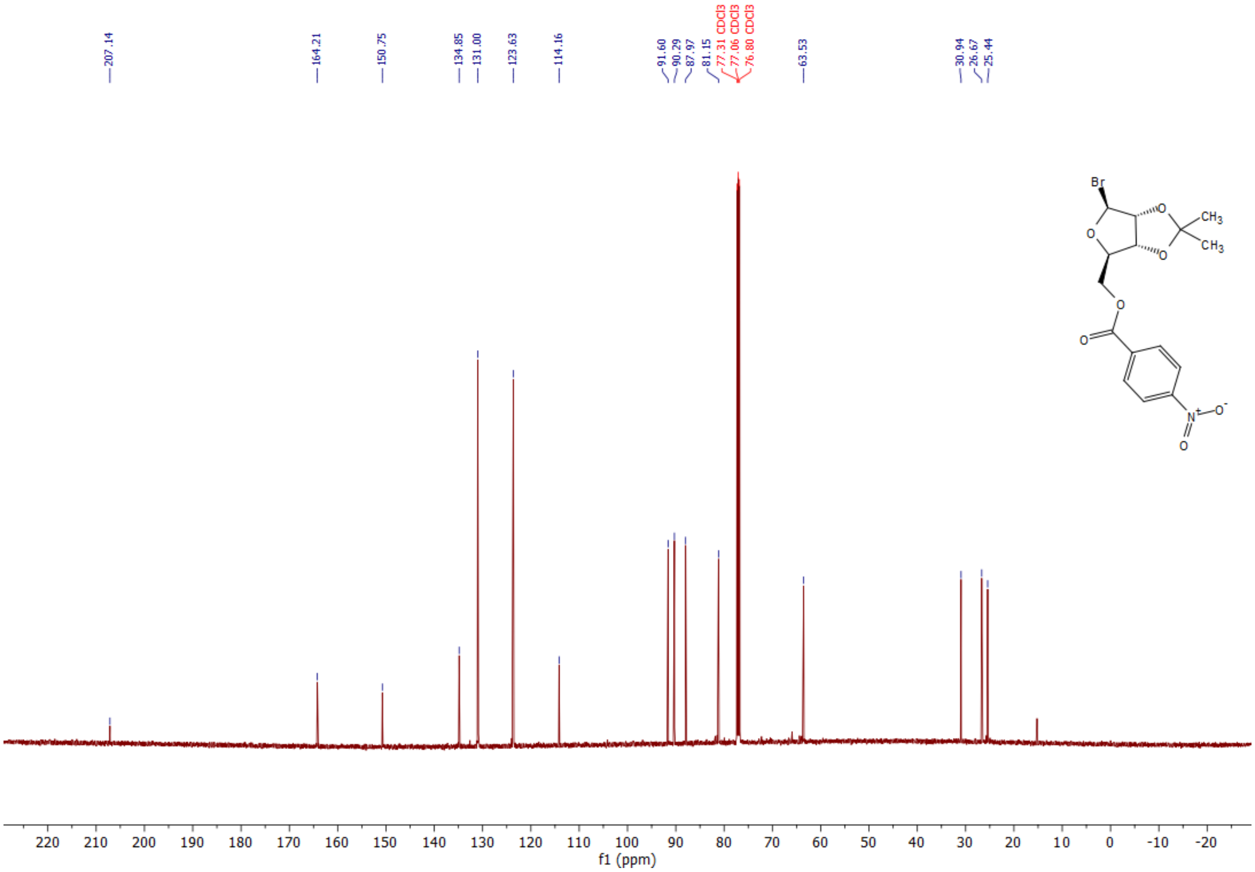

^{1}H NMR compound **4**

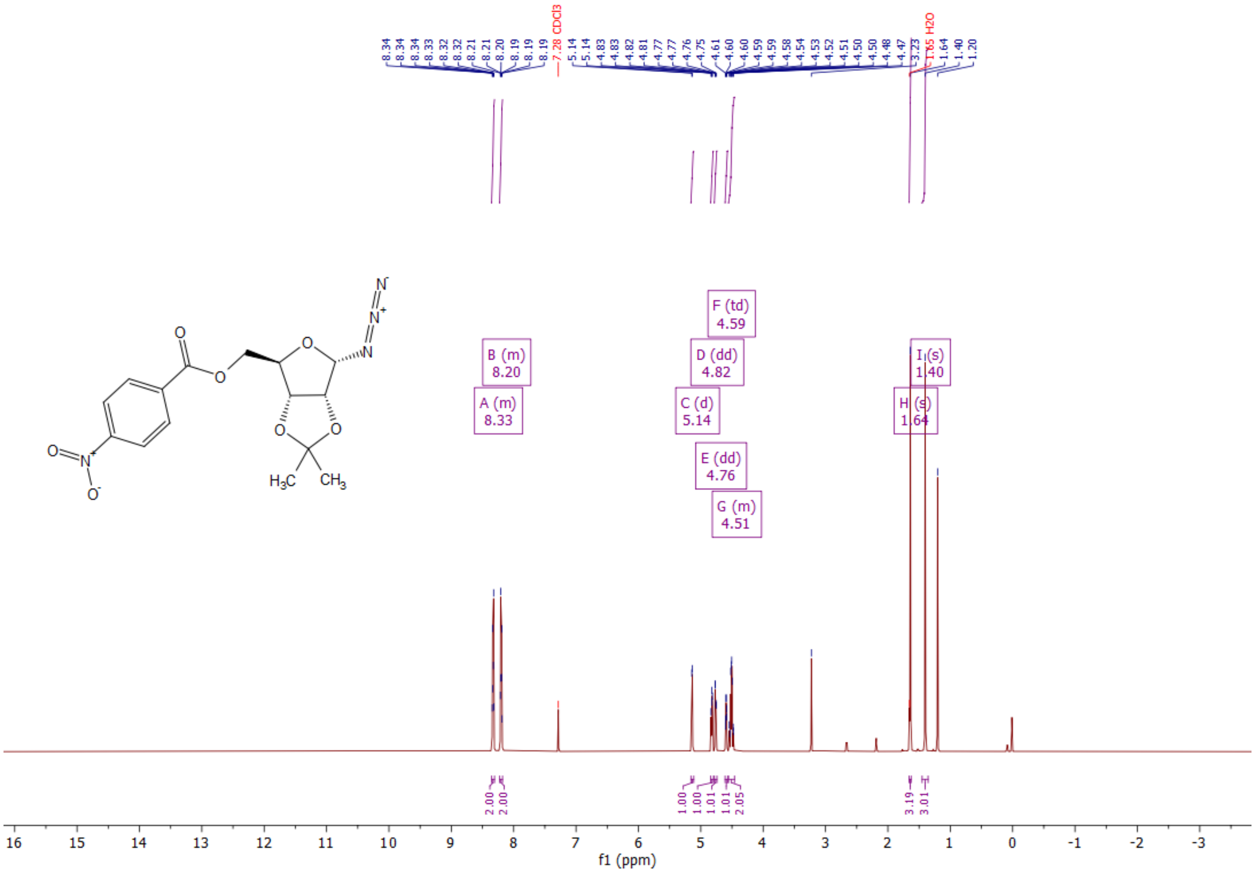

^{13}C NMR compound **4**

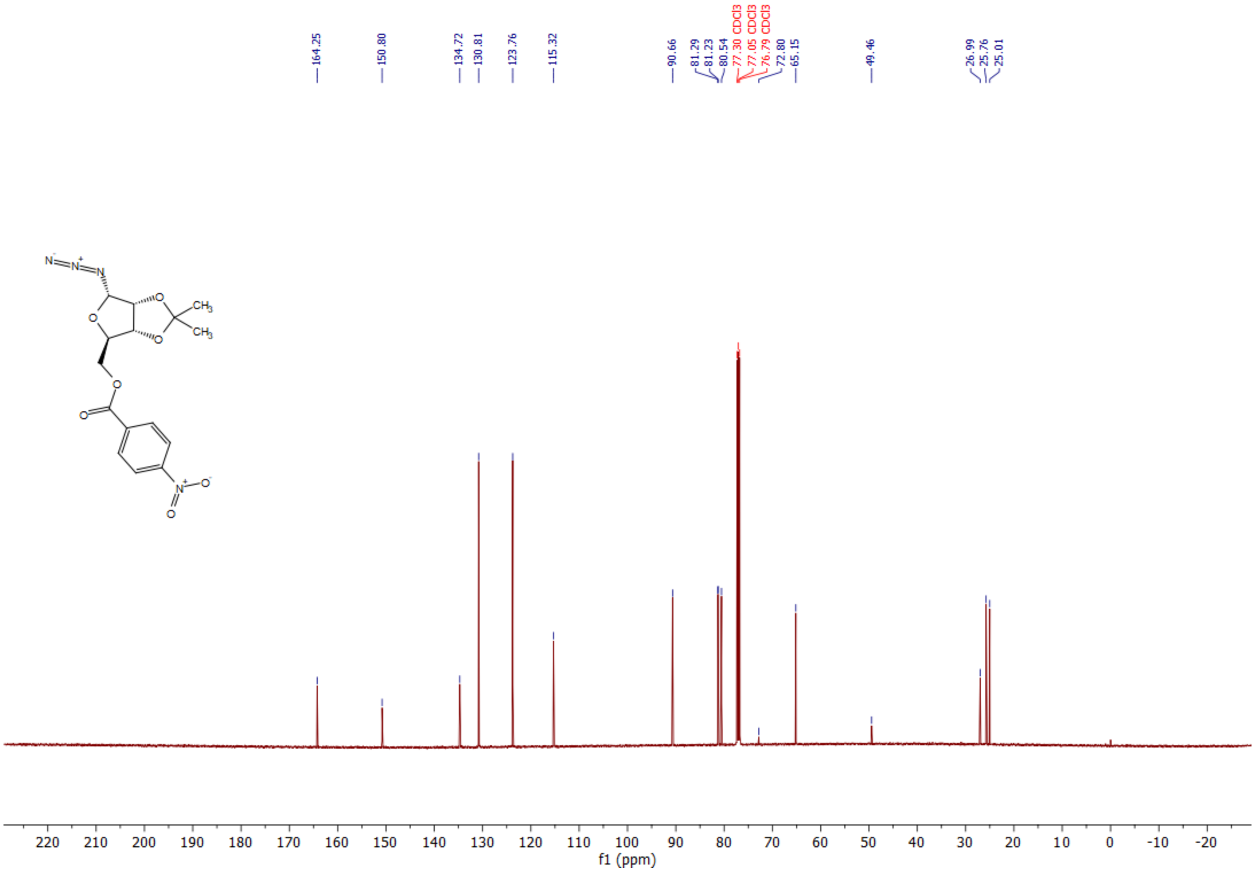

^{1}H NMR compound **5**

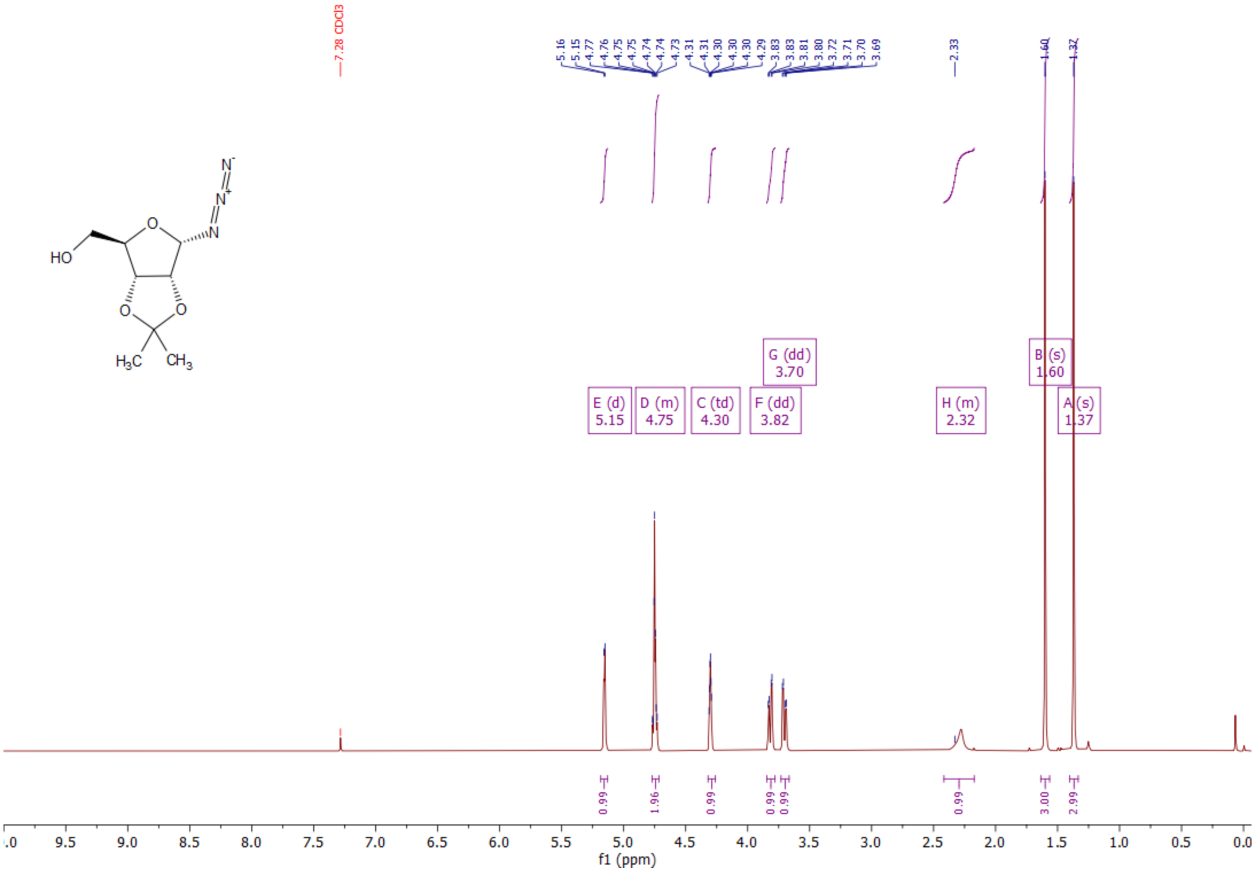

^{13}C NMR compound **5**

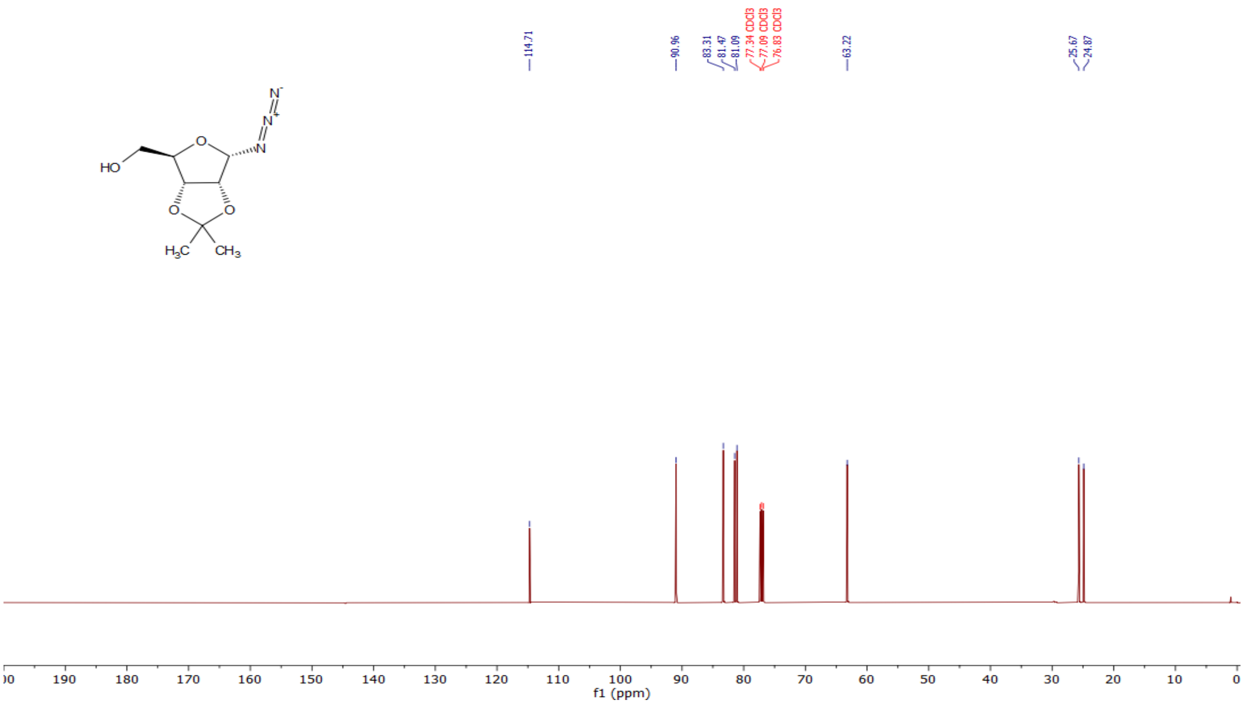

^{1}H NMR compound **6**

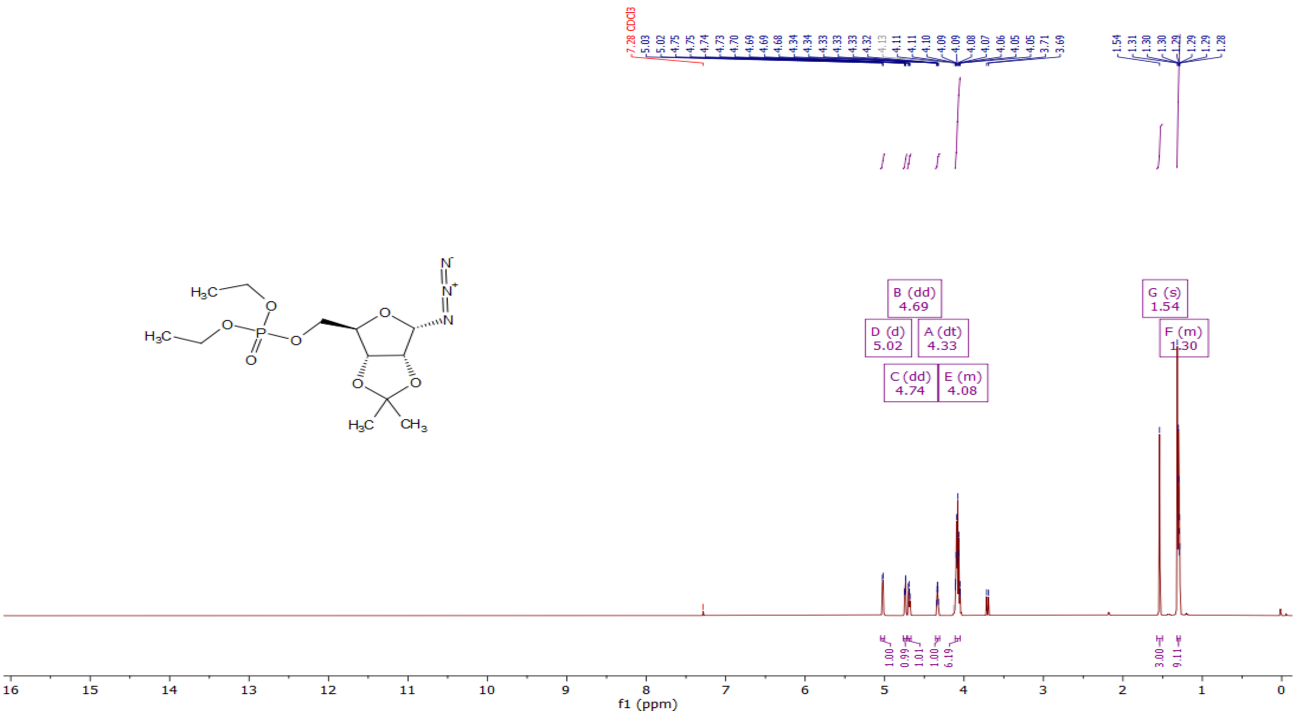

^{13}C NMR compound **6**

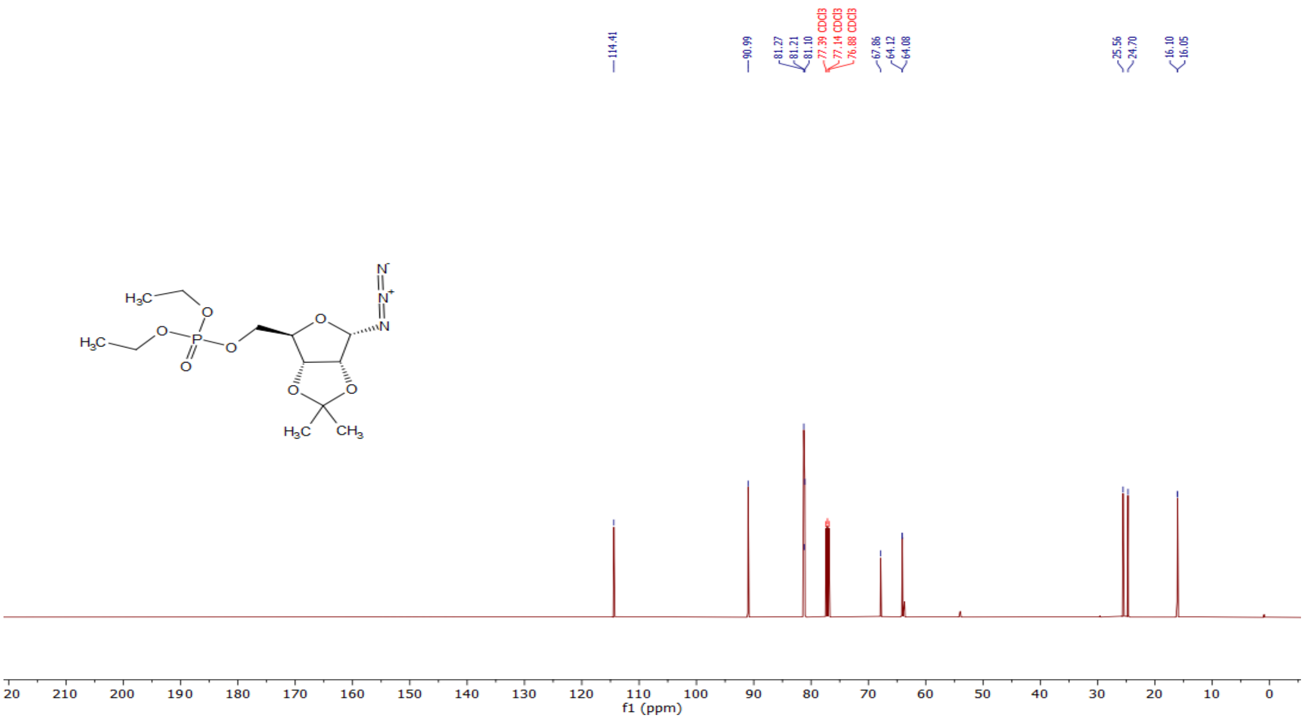

^{1}H NMR compound **7**

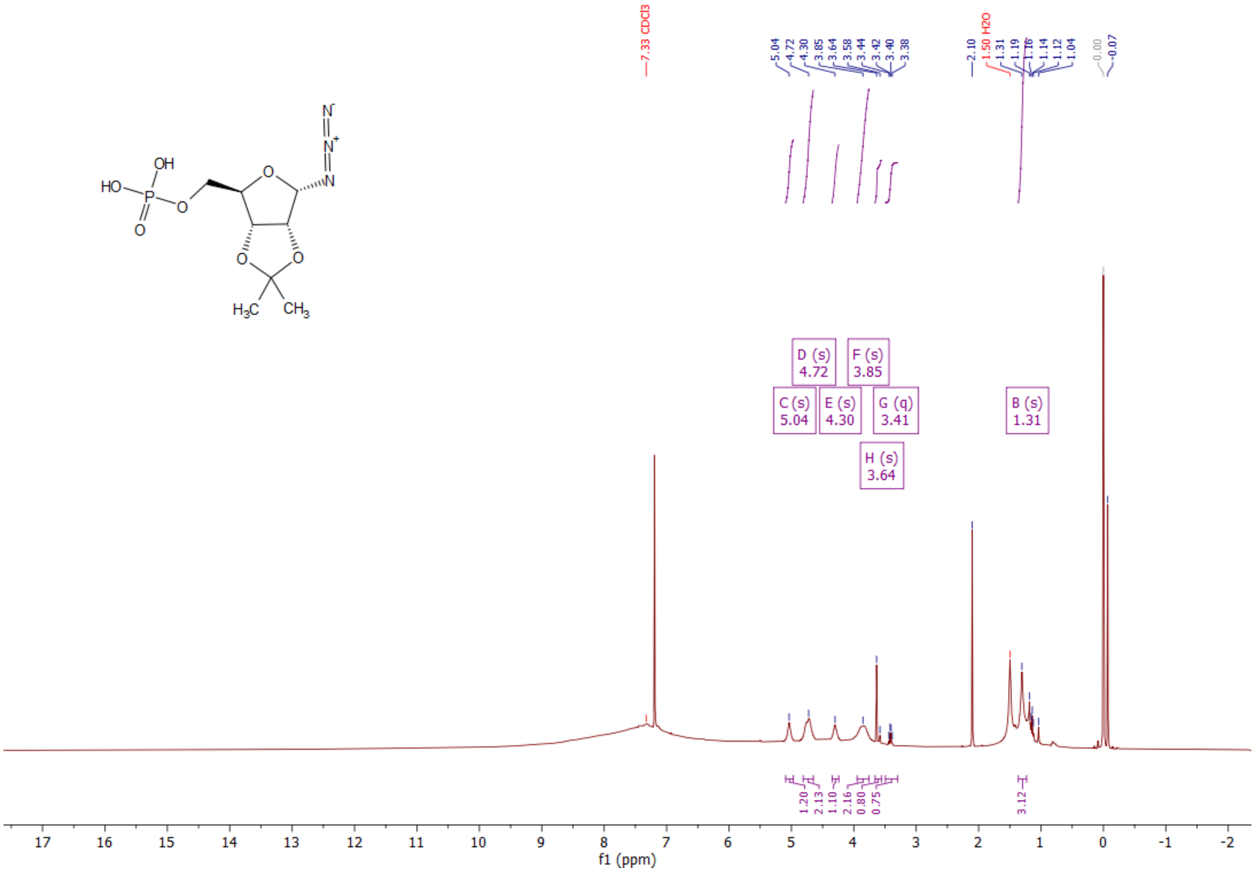

^{13}C NMR compound **7**

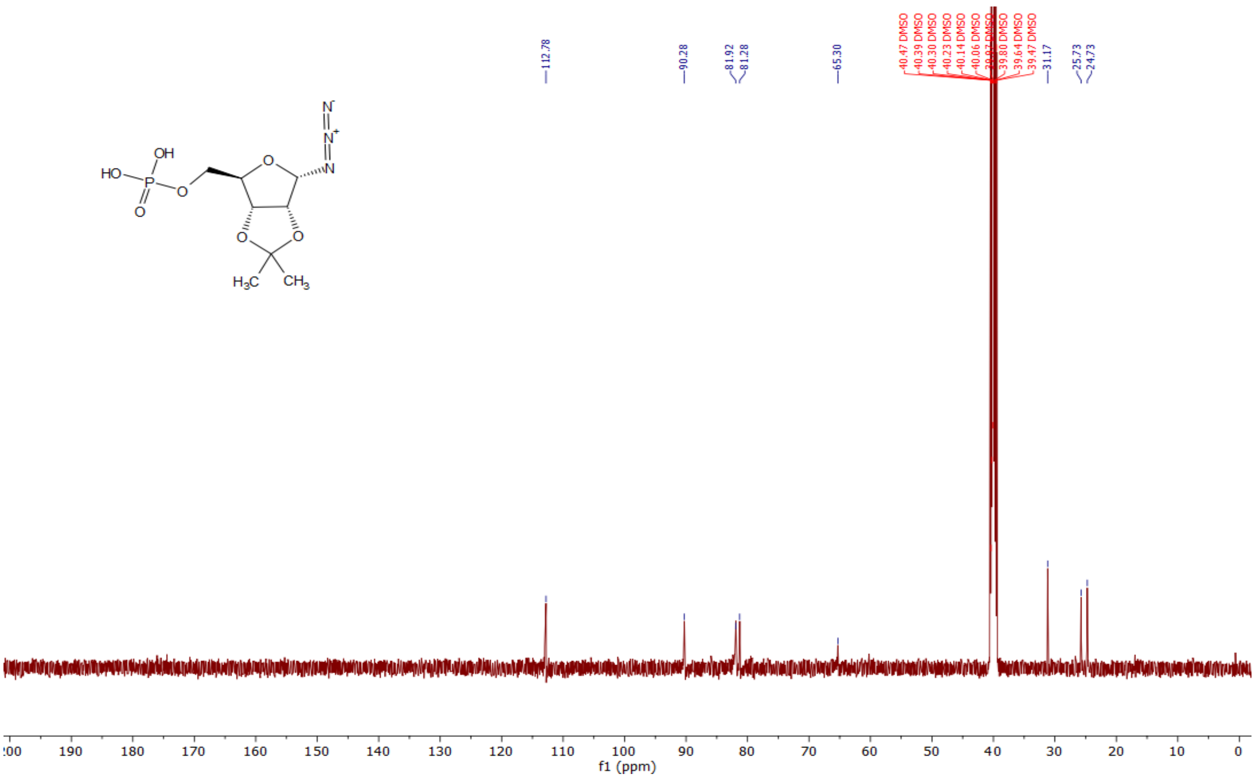

^{31}P NMR compound **7**

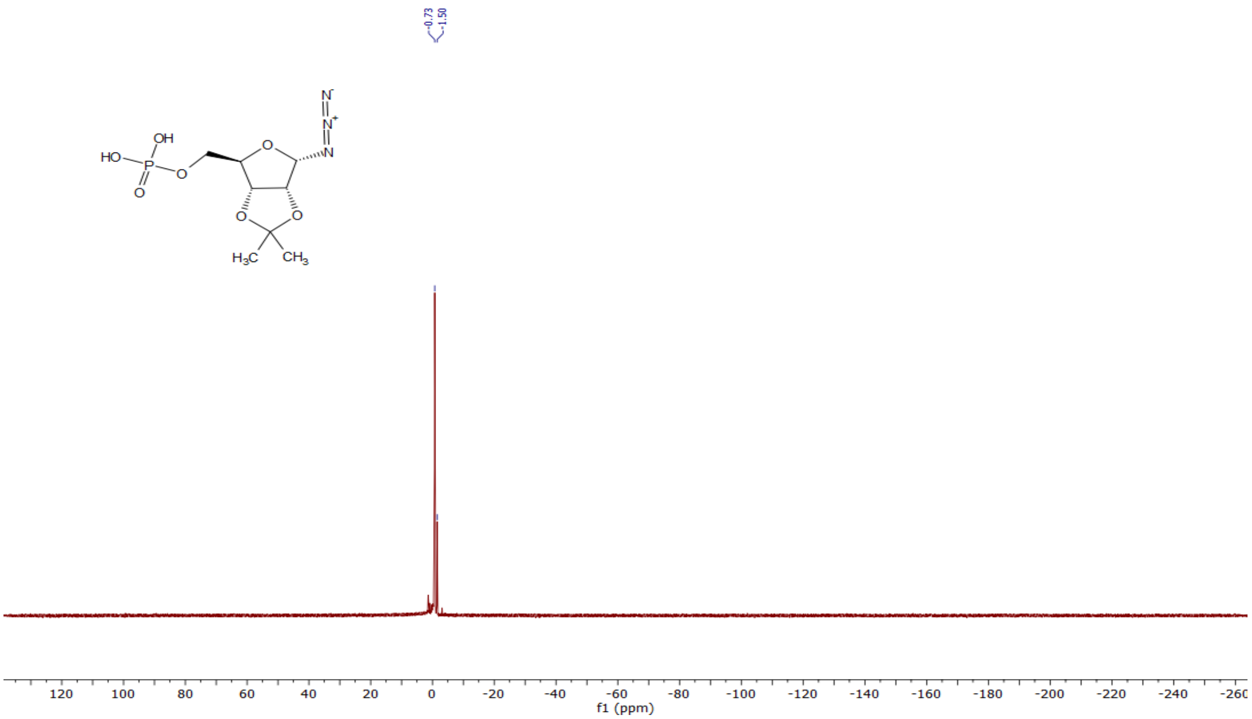

^{1}H NMR compound **10** (D_2O)

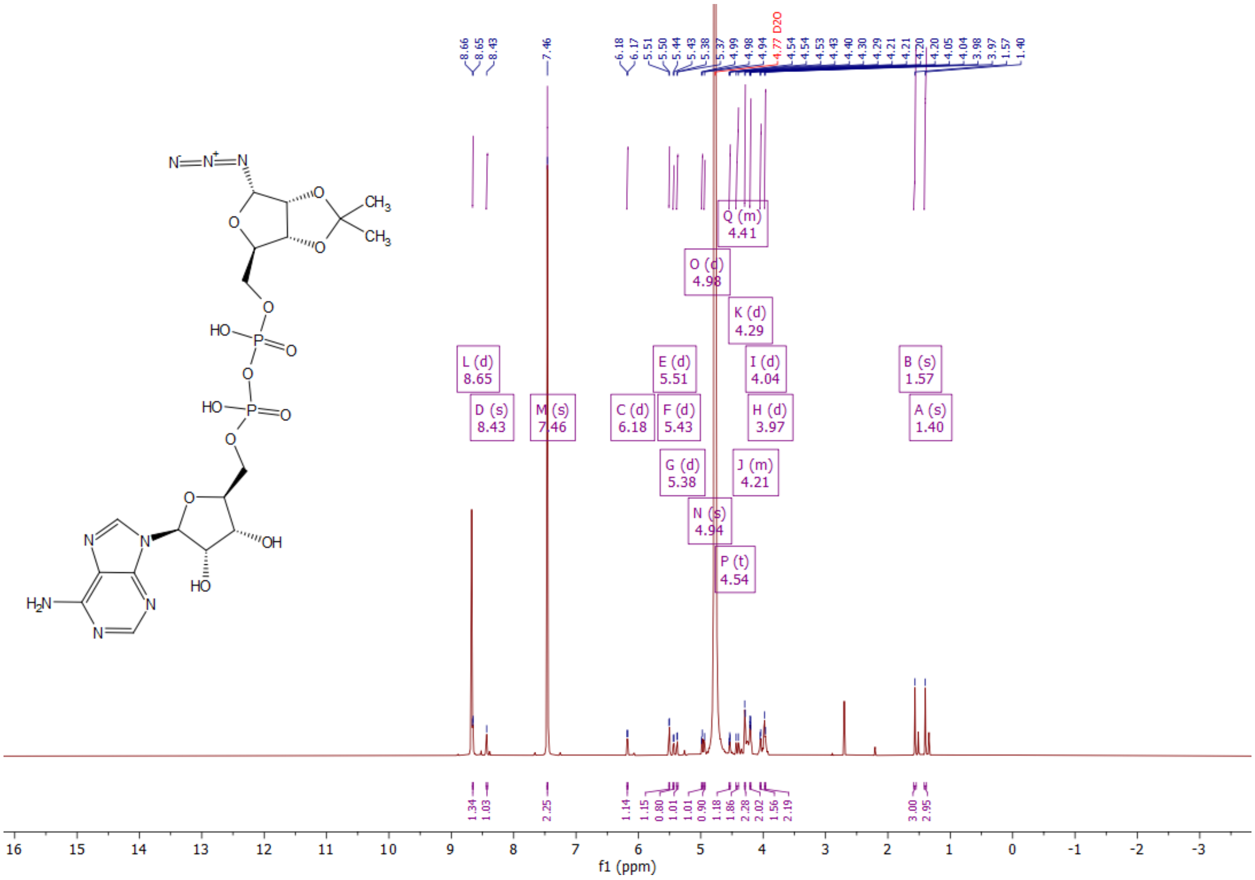

^{13}C NMR compound **10** (D_2O)

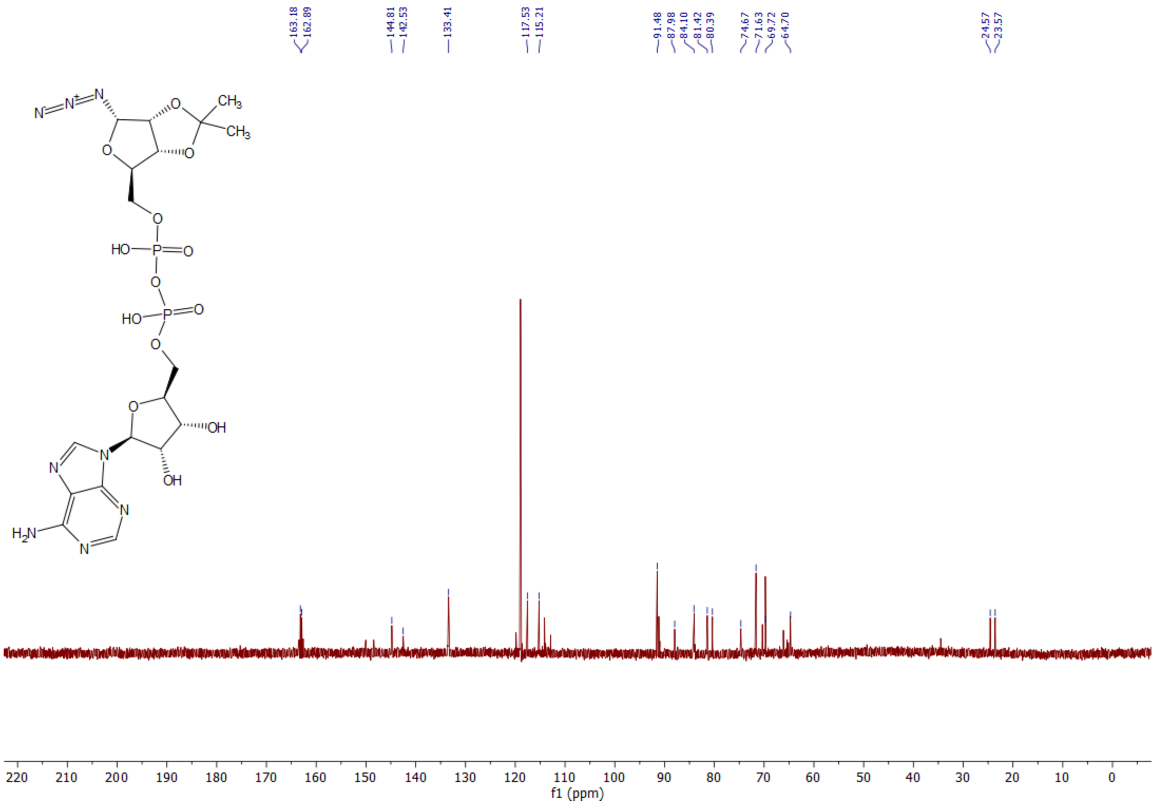

^{31}P NMR Compound **10**

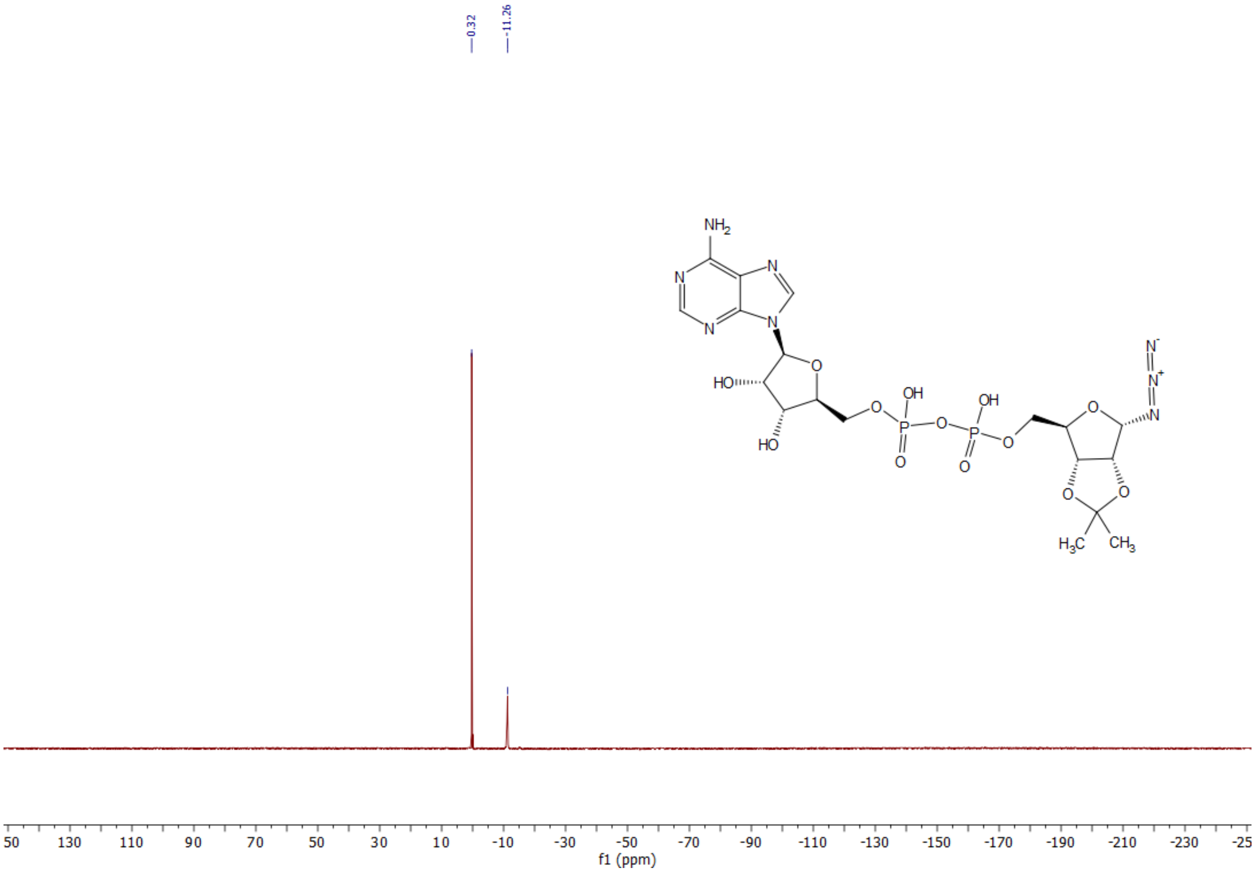

ADPr-N_3 (α)

^{1}H NMR compound **11 (α)**

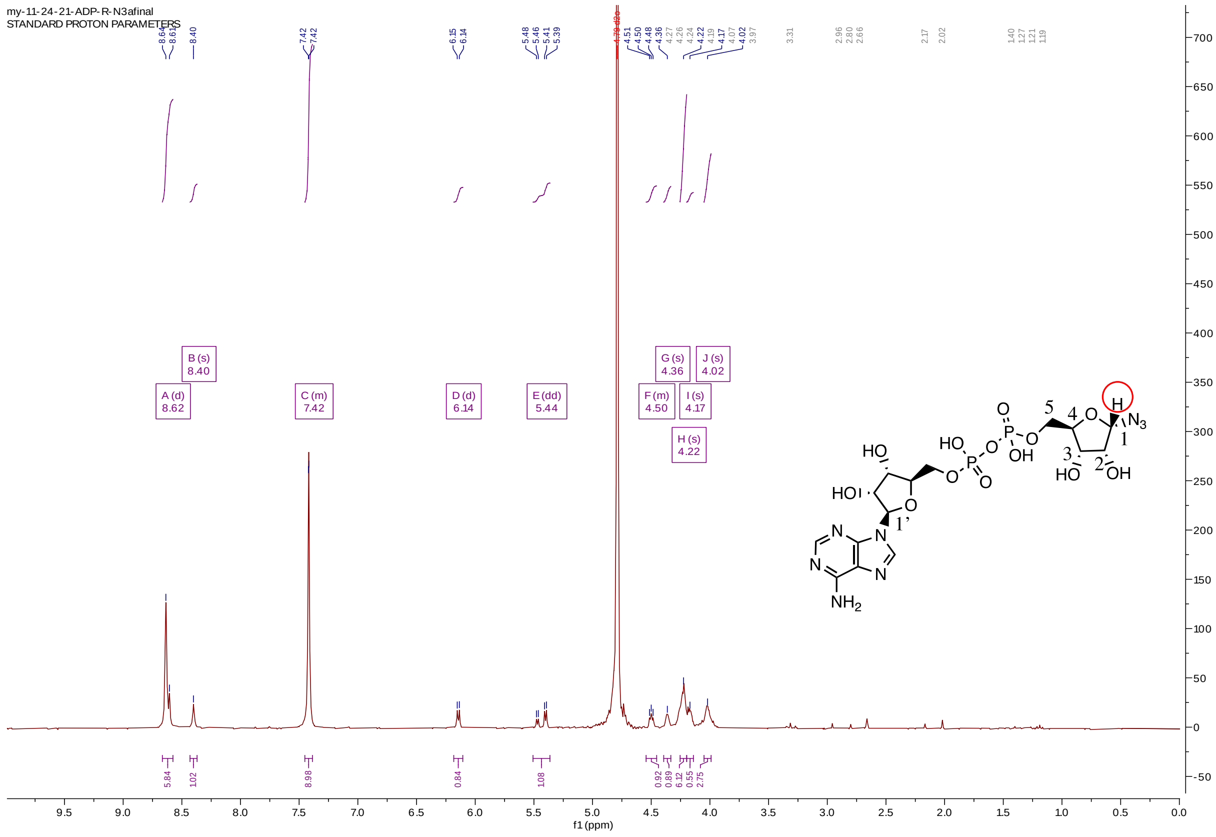

ADPr-N3 (β)

^{1}H NMR compound **11 (β)**

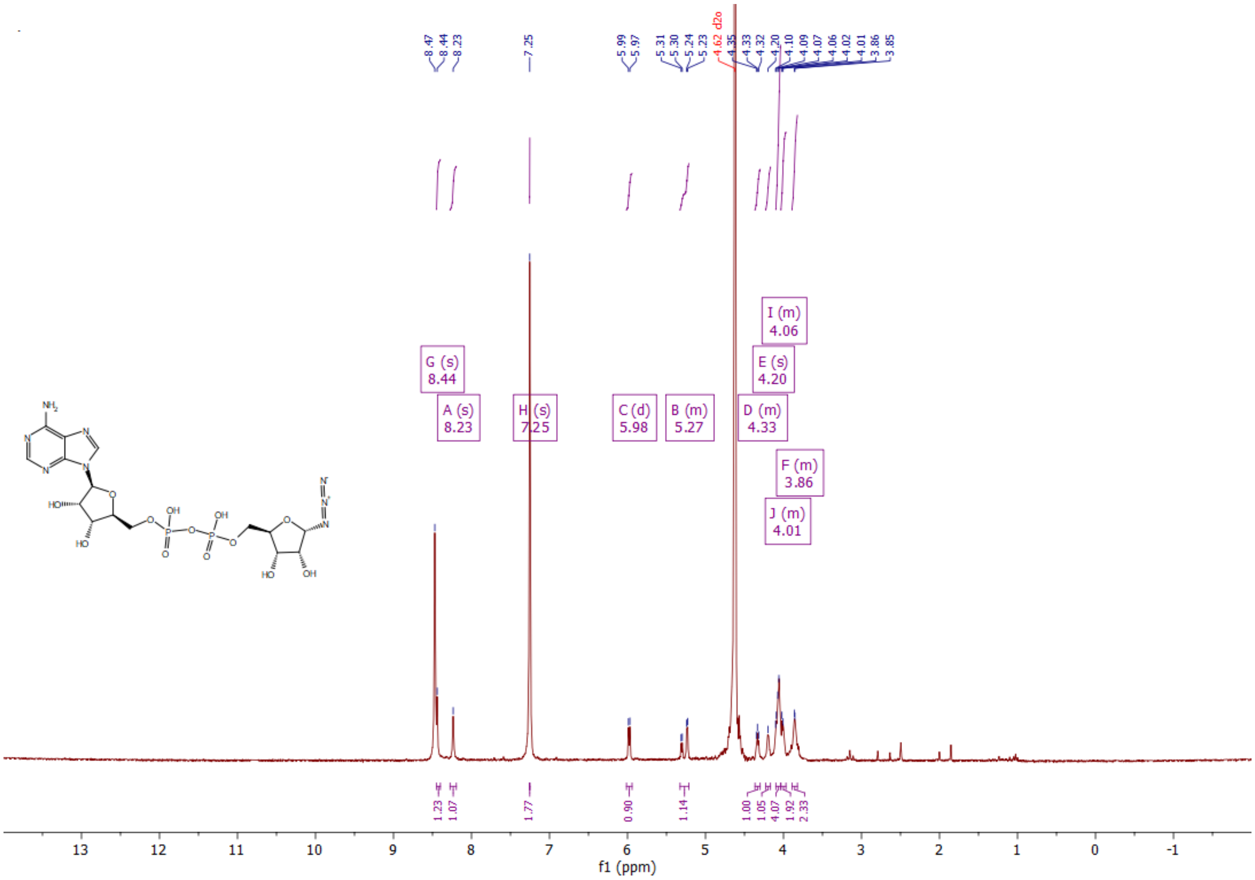

^{13}C NMR compound **11**

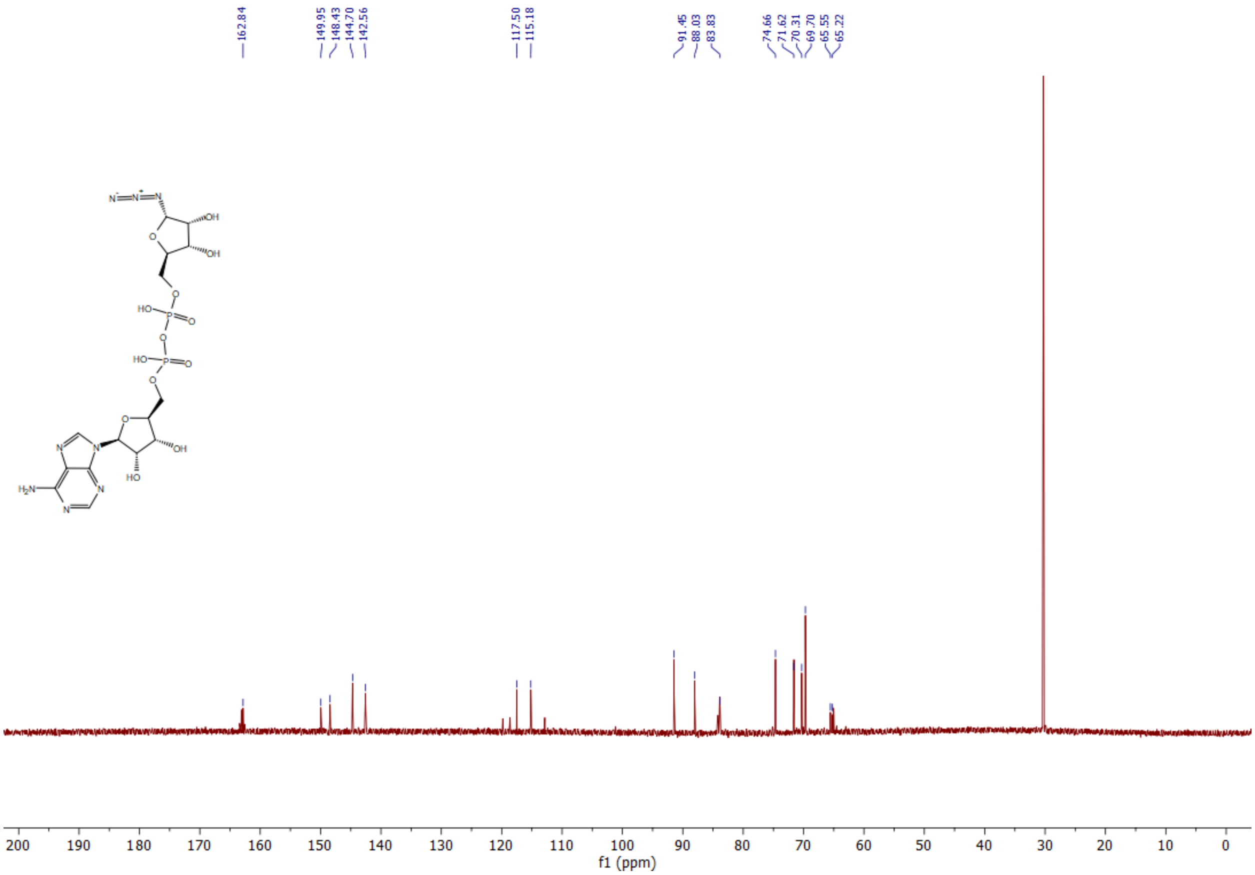

^{31}P NMR compound **11**

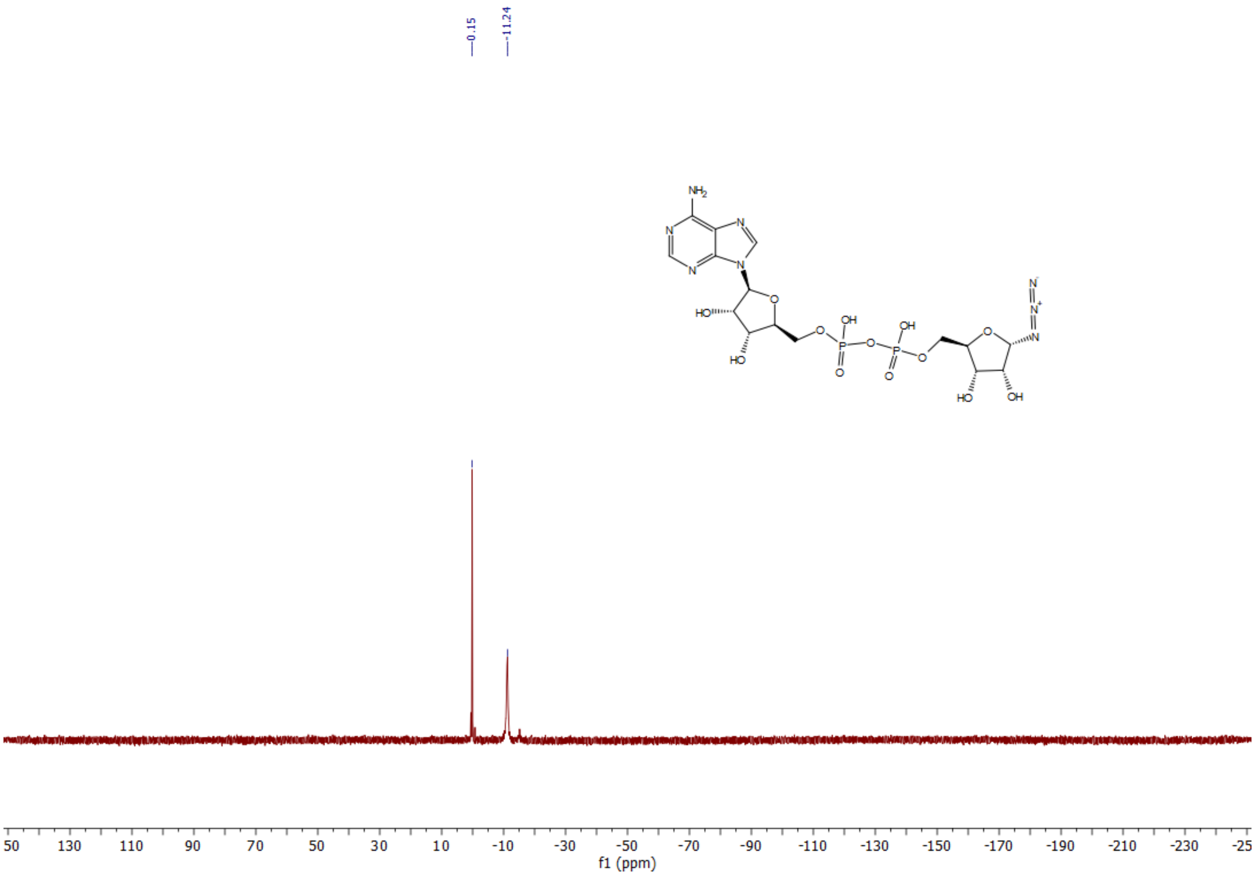

HPLC purification of **compound 11**

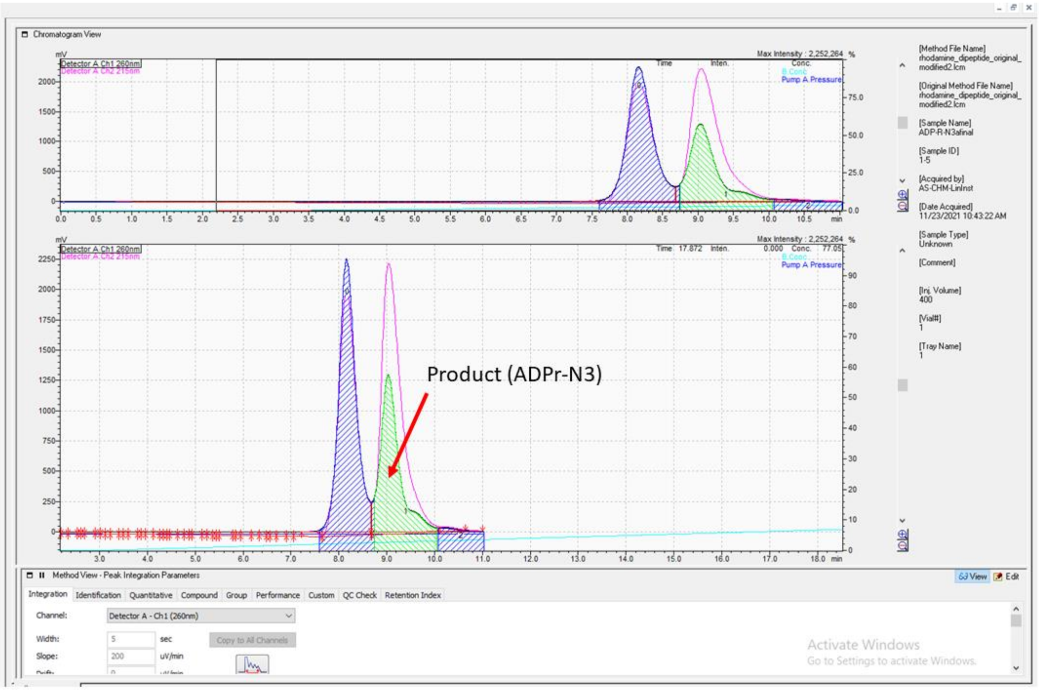

^{1}H NMR compound **12**

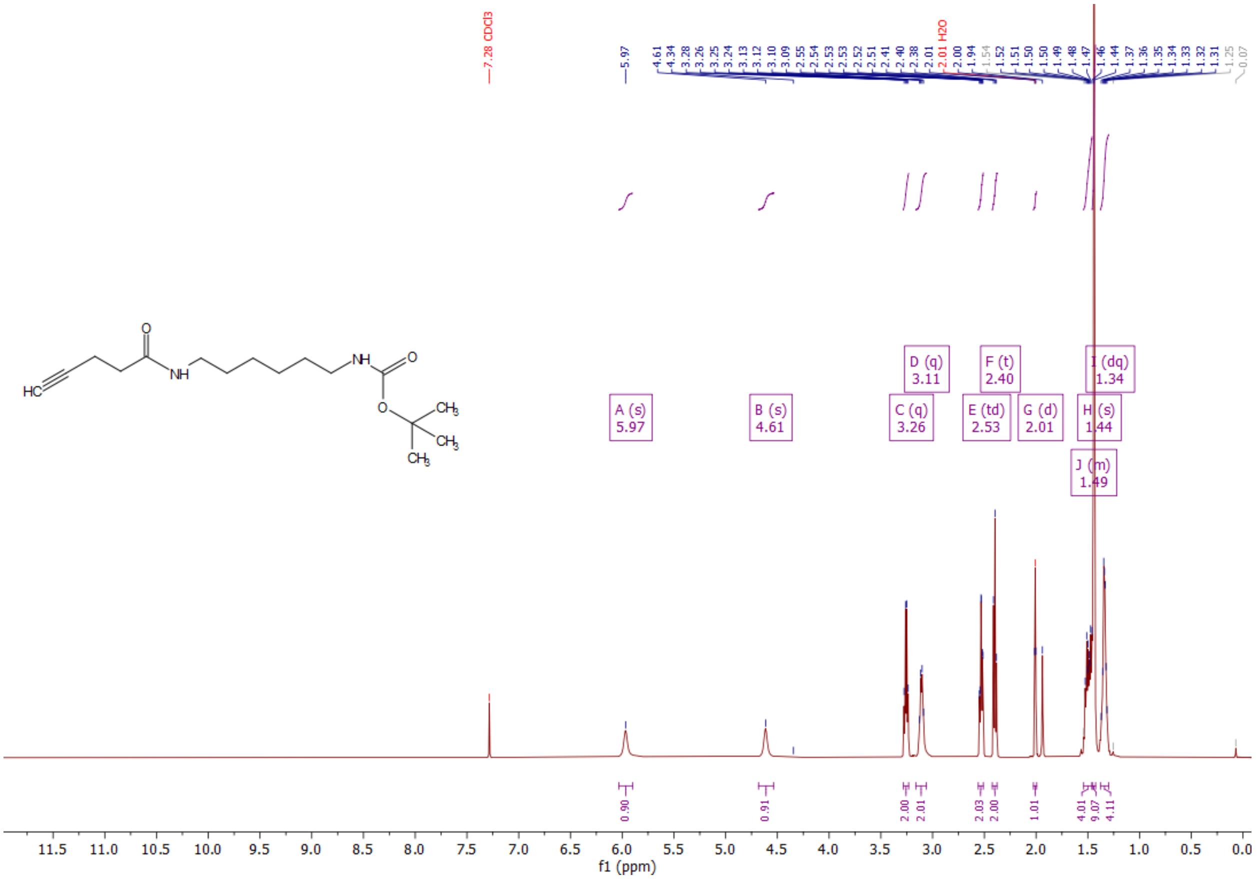

^{13}C NMR compound **12**

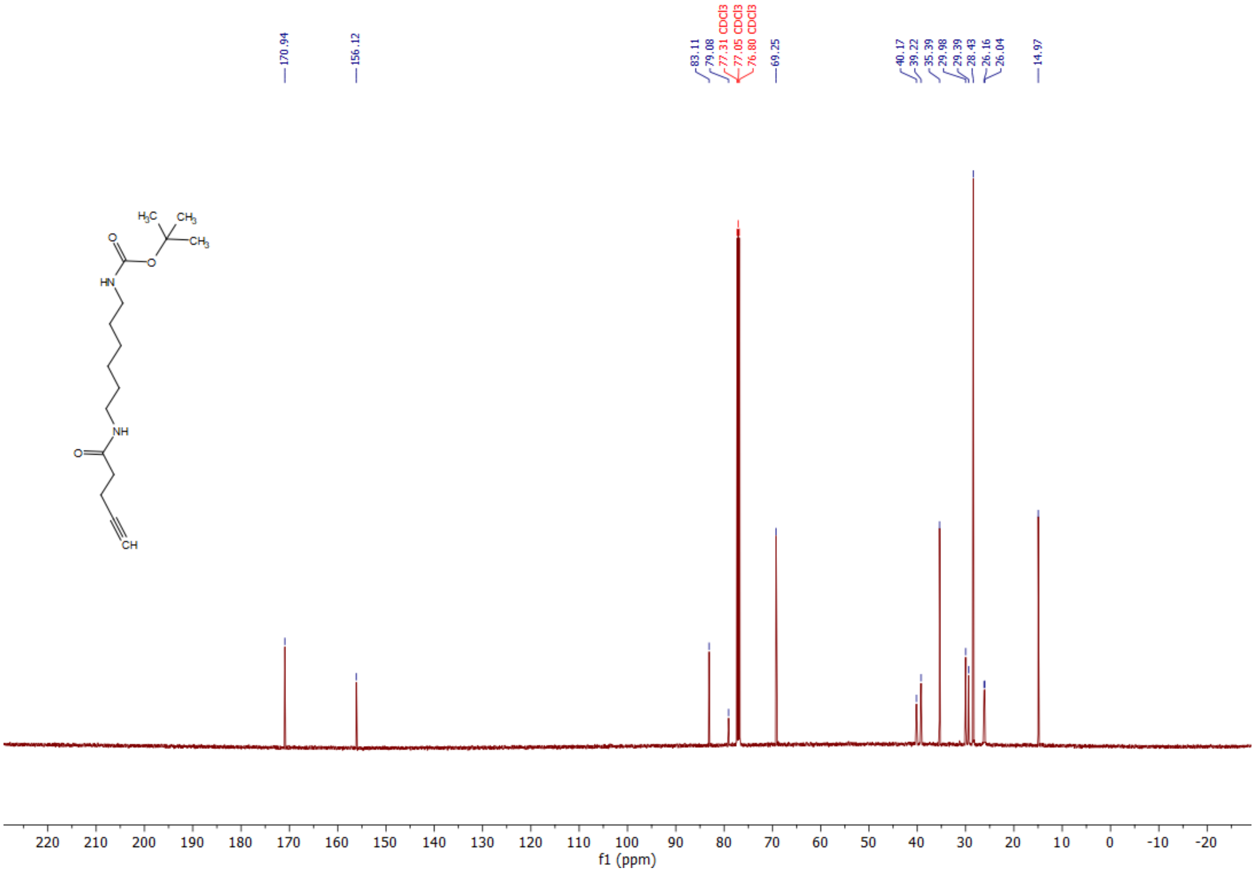

^{1}H NMR compound **14**

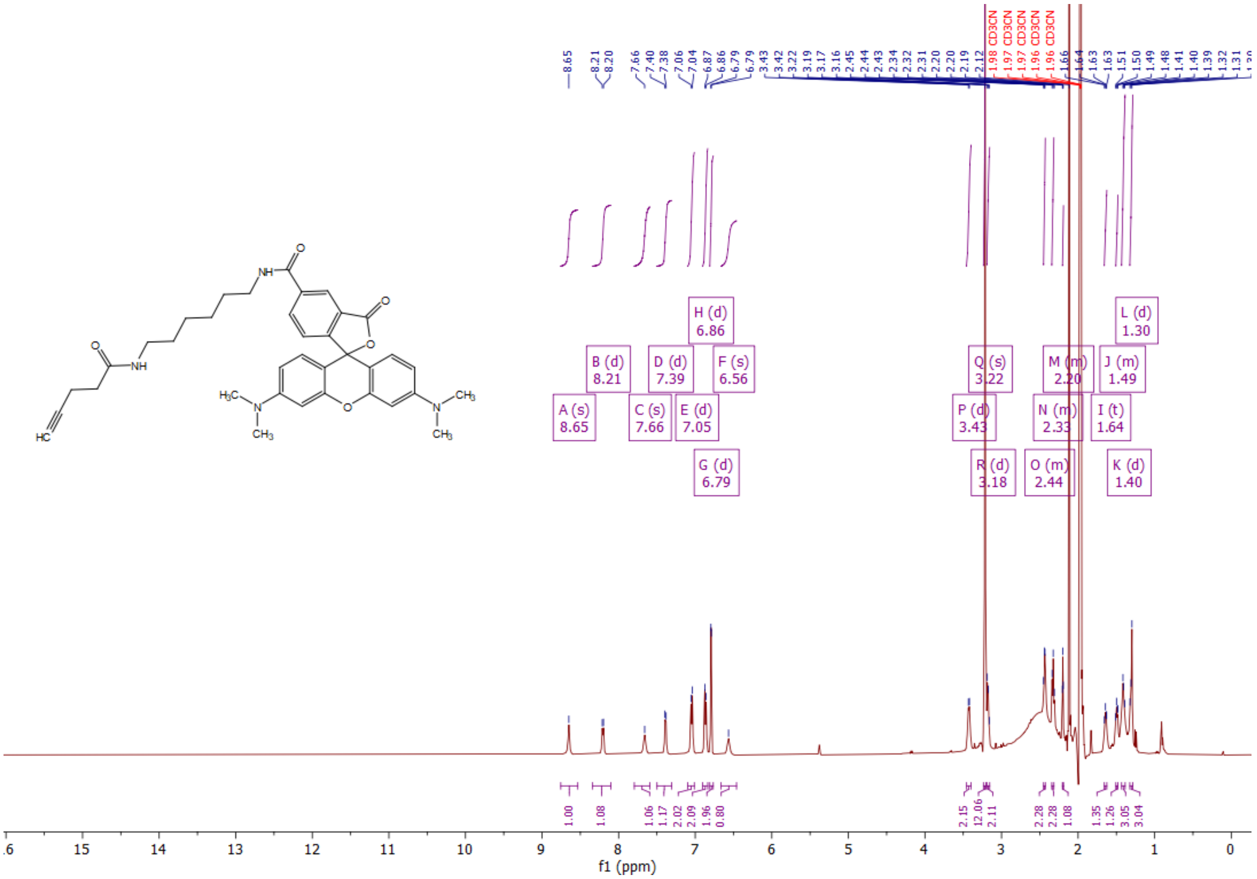

^{13}C NMR compound **14**

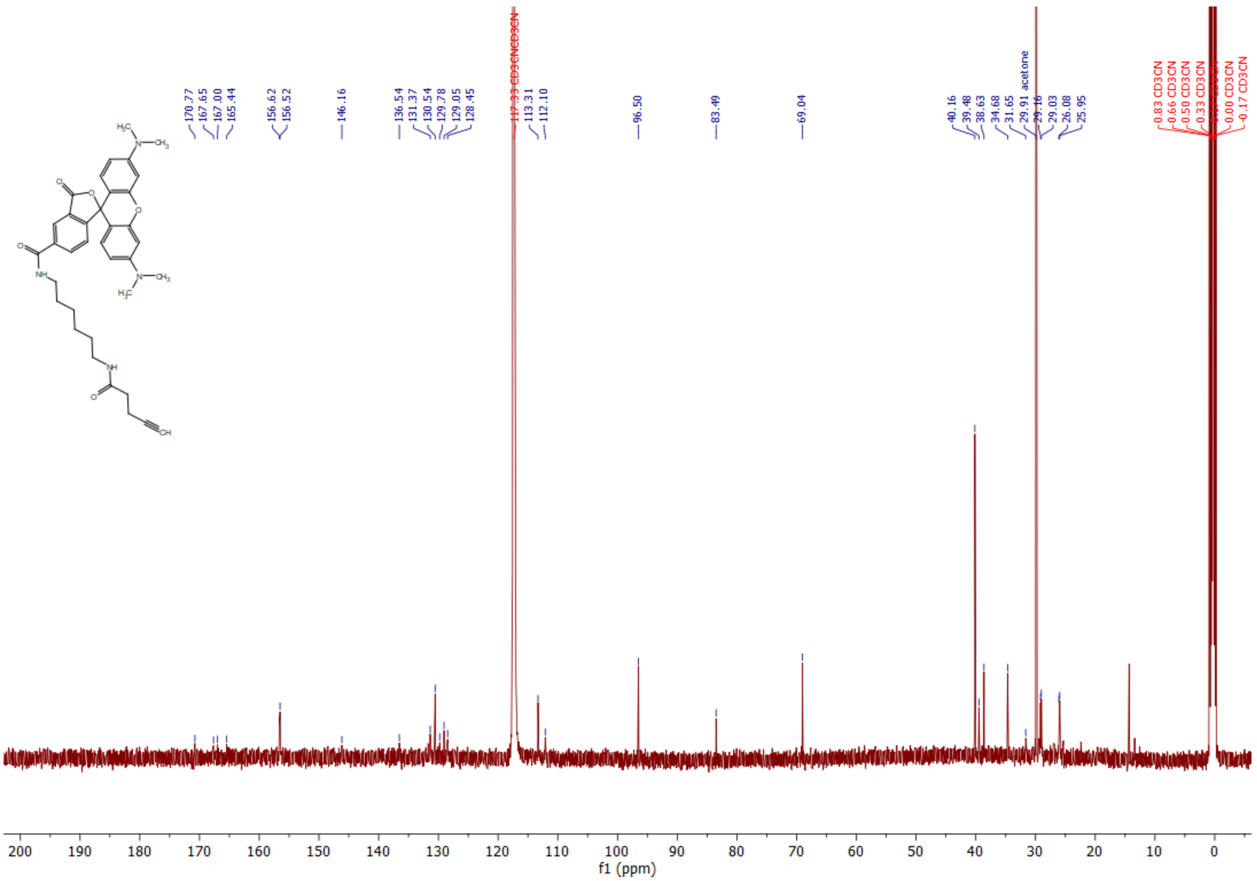

TAMRA-ADPr ^{1}H NMR

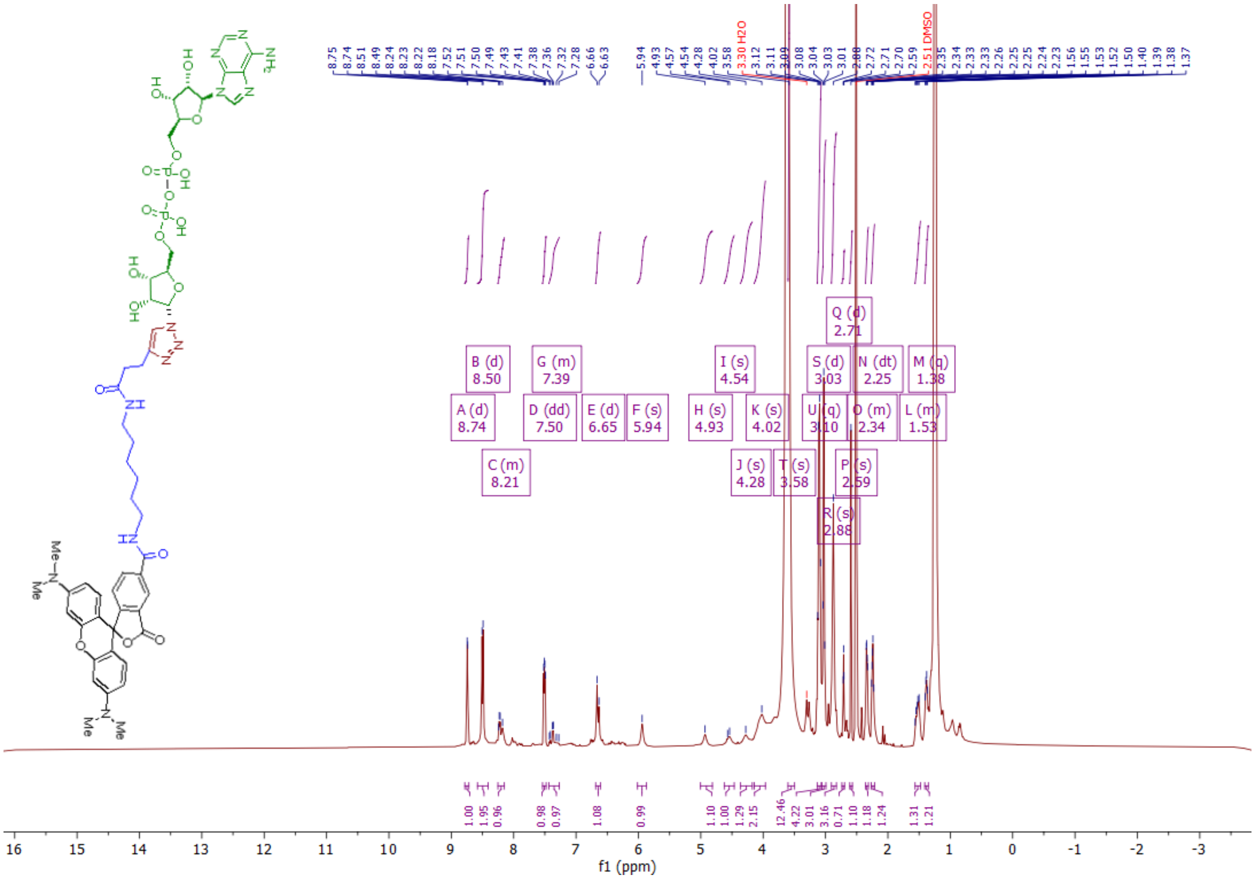

^{13}C NMR

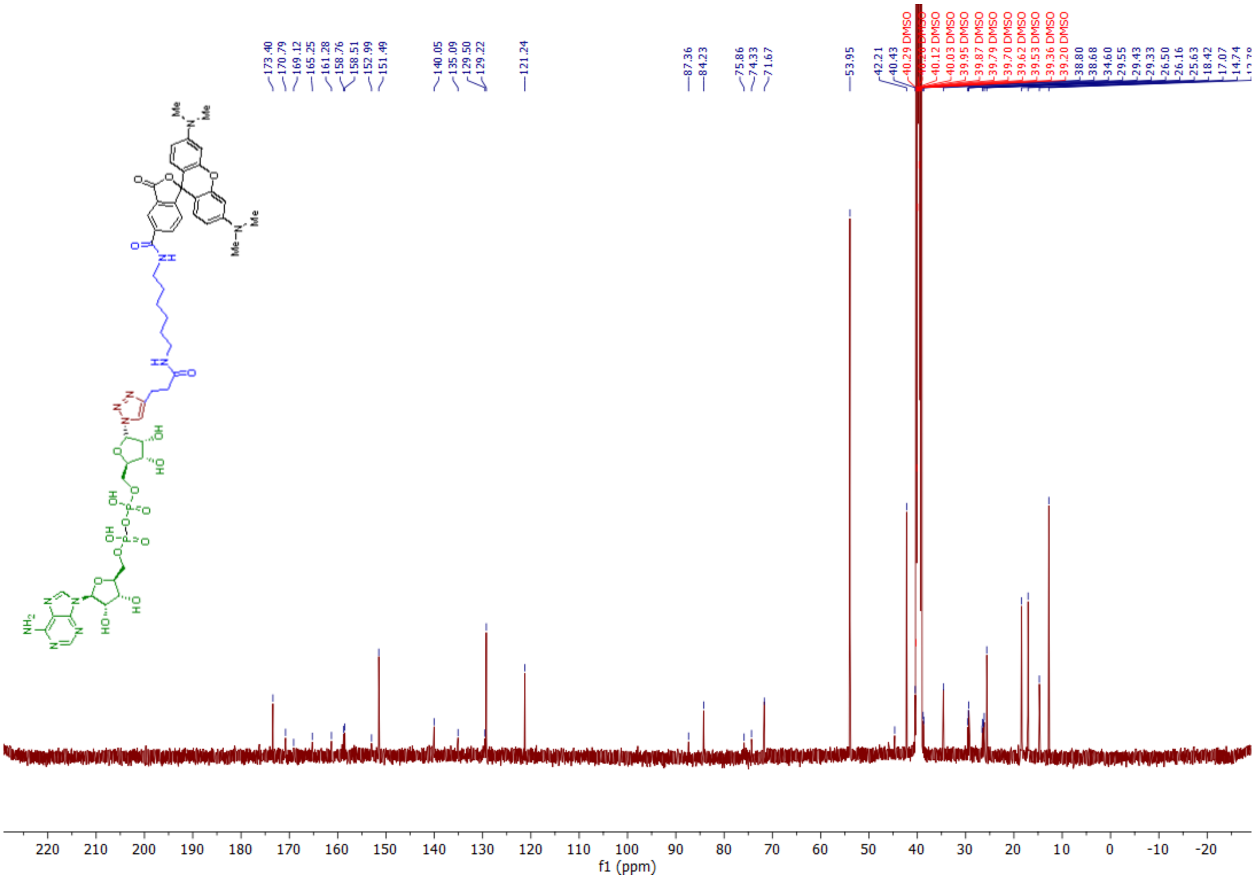

^{31}P NMR

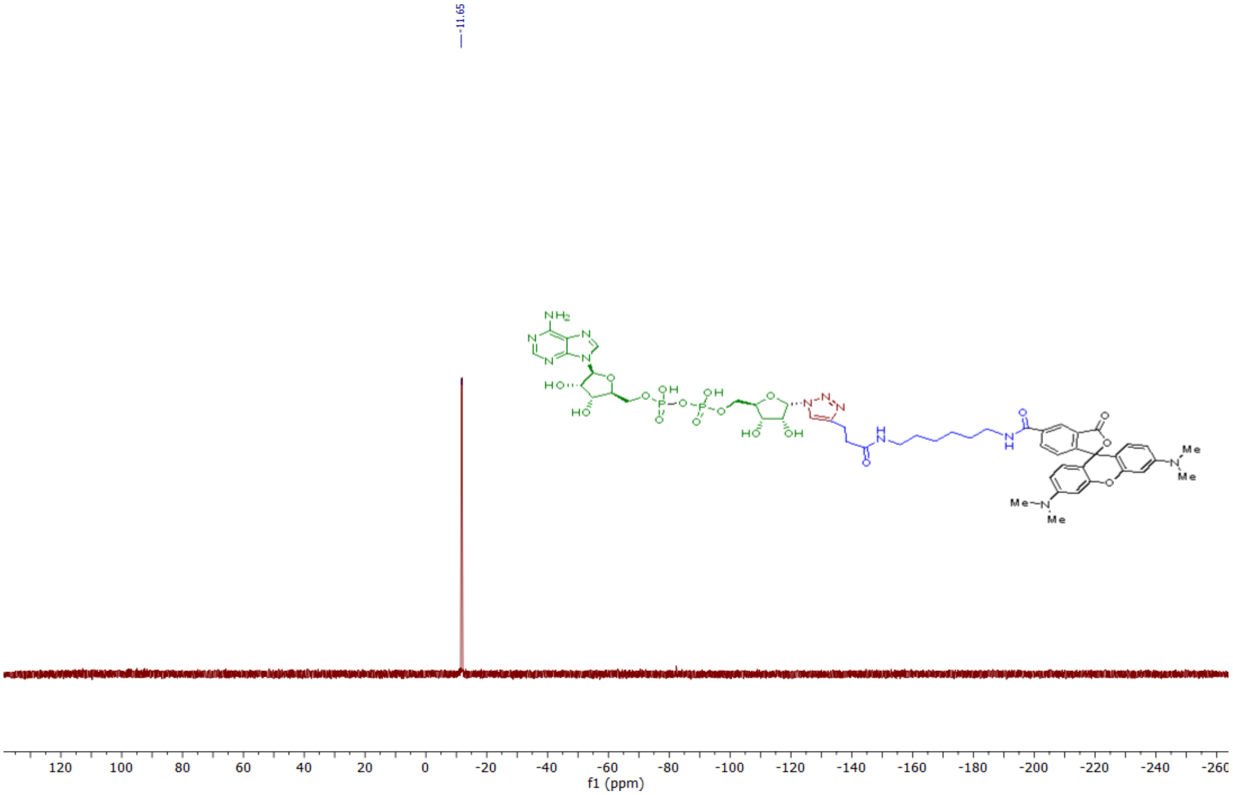

Reaction mixture LCMS:

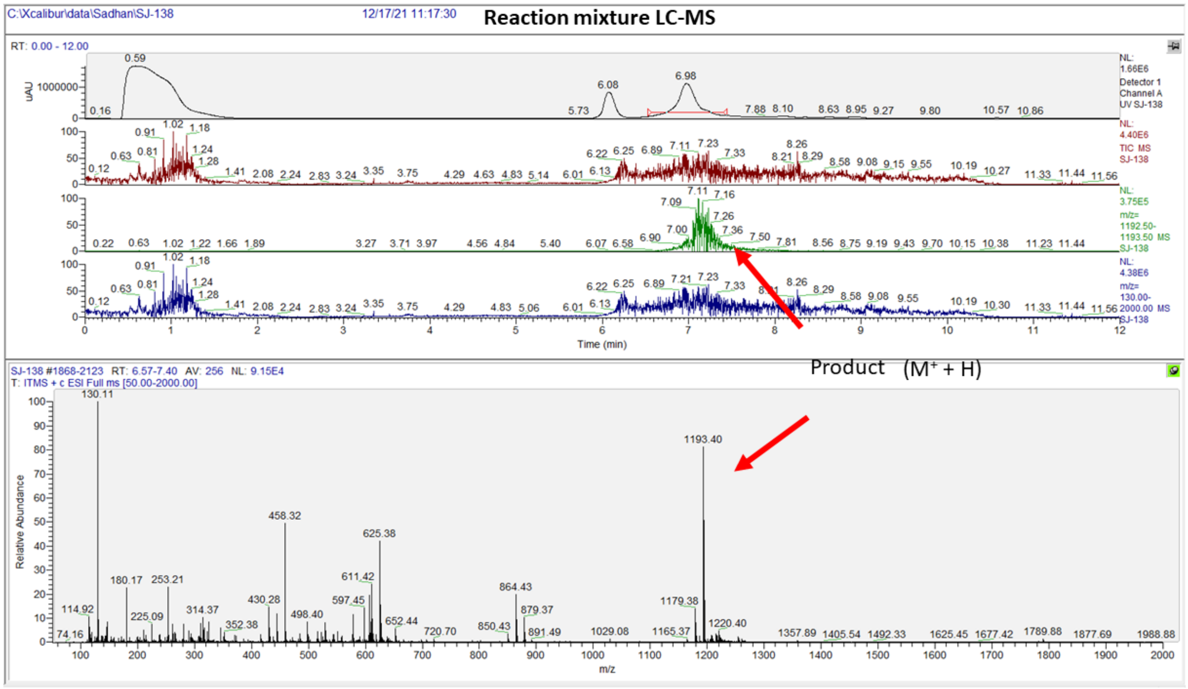

HPLC - Purification

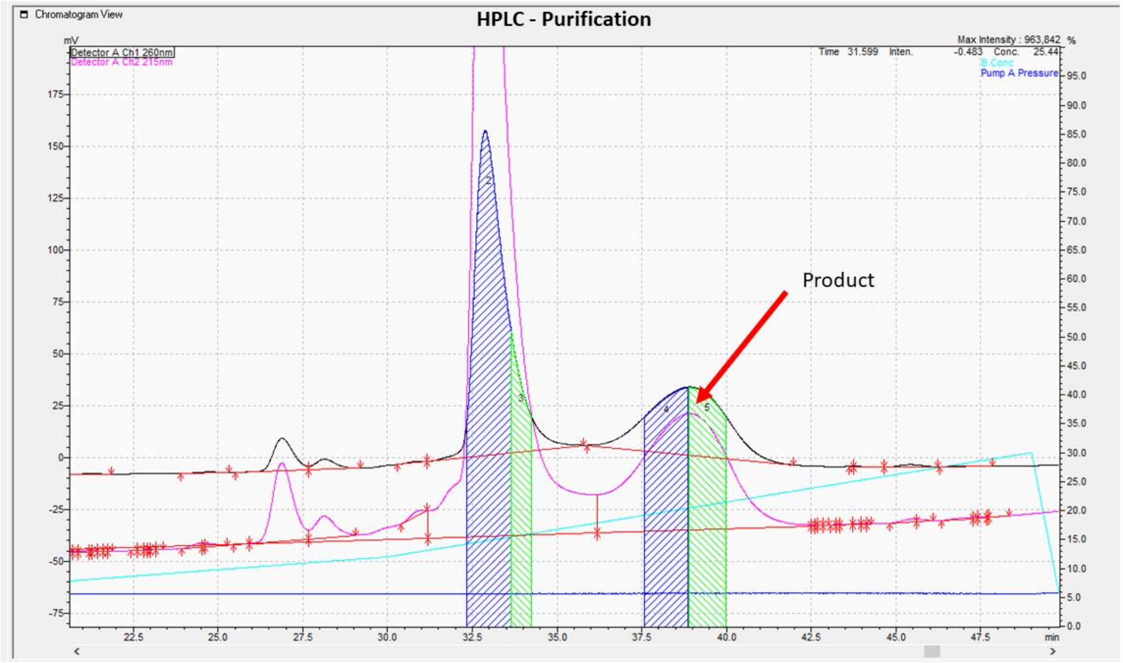

LCMS analysis of HPLC-purified Probe

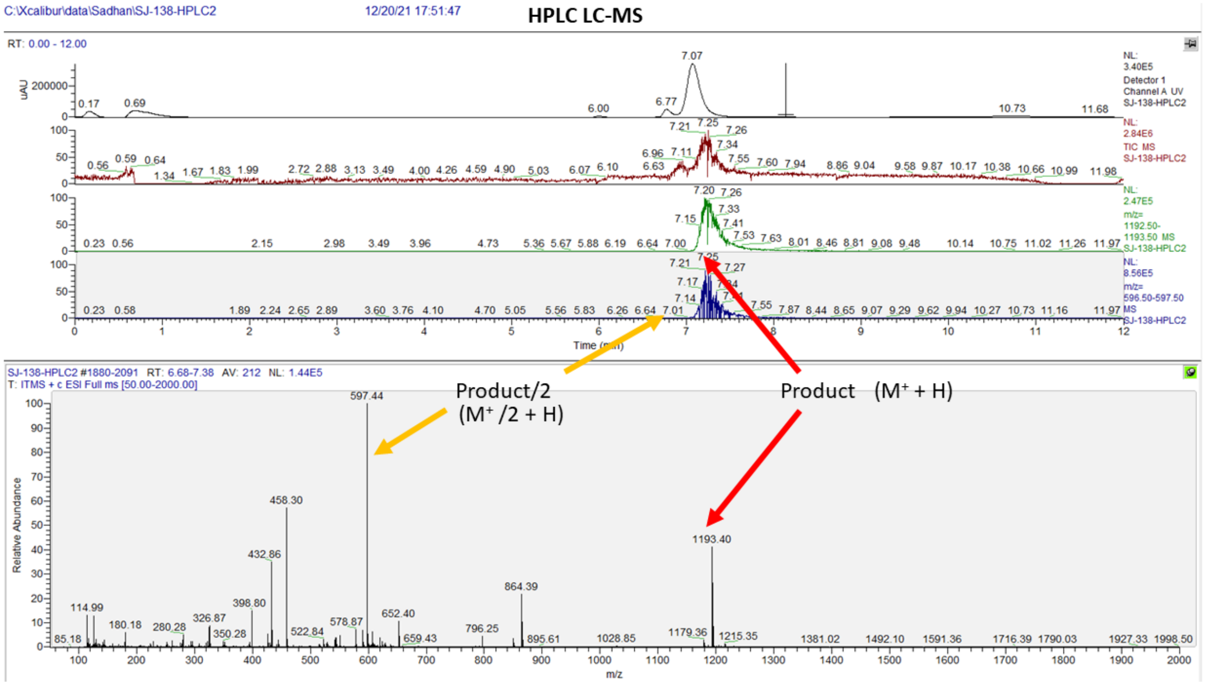

LCMS: Thermo Scientific LTQ XL.

Column: P/No. 00A-4633-AN, Kinetex^R, 5 μm EVO, C18, 100 Å; LC column 30 x 21 mm.

Solvent A: water with 0.1% formic acid; **Solvent B**: acetonitrile with 0.1% formic acid.
